# Analysis of Volatile Secondary Metabolites in *Ocimum basilicum* Cell Suspensions: Inhibition, In Silico Molecular Docking, and an ADMET Analysis against Proteolytic Enzymes of *Rhynchophorus ferrugineus*

**DOI:** 10.3390/plants11212949

**Published:** 2022-11-01

**Authors:** Hossam Moustafa Darrag, Hani Taher Almuhanna, Emadaldeen Hamad Hakami, Sameer M. Alhojaily

**Affiliations:** 1 Department of Research and Training, Research and Training Station, King Faisal University, Al-Ahsa 31982, Saudi Arabia; 2 Pesticide Chemistry and Technology Department, Faculty of Agriculture, Alexandria University, Alexandria 21545, Egypt; 3 Research and Training Station, King Faisal University, Al-Ahsa 31982, Saudi Arabia; 4 Department of Biomedical Sciences, College of Veterinary Medicine, King Faisal University, Al-Ahsa 31982, Saudi Arabia

**Keywords:** red palm weevil, eco-insecticides, chemical composition, LS liquid media, somatic embryogenesis, target proteolytic enzyme

## Abstract

Our study’s overarching goal was to determine which *O. basilicum* cell suspensions approach yielded the most insecticidal and *R. ferrugineus*-inhibitory volatile secondary metabolites. After inoculation with *Verticillium dahliae* as an activator, the growth kinetics were measured, and the extract was identified using GC-MS. Validation was achieved for the insecticidal efficacy of a volatile extract, the pure phenolic content against larva and adult *R. ferrugineus*, and the inhibitory effect on proteases (in vivo and in vitro). The volatile extract achieved an LC_50_ of 1229 µg/mL and an LD_50_ of 13.8 µg/larva. The LC_50_ values for β-bergamotene, α-eudesmol, β-farnesene, linalool, 1,8-cineole, eugenol, α-guaiene, and β-caryophyllene were 1294, 1312, 1356, 1398, 1426, 1459, 1491, and 1523 g/mL, respectively. The LD_50_ activities of α-eudesmol, linalool, 1,8-cineole, eugenol, and nerol were 12.4, 13.7, 13.9, 14.2, and 15.6 g/larva, respectively. Active volatile extract of *O. basilicum* inhibited trypsin proteinase, elastase, cysteine, overall protease, and metalloprotease activity with IC_50_ values of 89.4, 101.7, 394.7, 112.4, and 535.2 µg/mL and 178.5, 192.4, 547.3, 208.3, and 924.8 µg/mL, in vitro and in vivo, respectively. There was evidence of action against total proteases (in vitro) with IC_50_ values of 78.9, 81.2, 88.6, 90.7, 91.5, 97.6, 107.4, and 176.3 µg/mL for β-bergamotene, α-eudesmol, β-farnesene, linalool, 1,8-cineole, eugenol, α-guaiene, and β-caryophyllene, respectively. Total proteases (in vivo) are inhibited by the α-eudesmol, linalool, 1,8-cineole, eugenol, nerol, and (E)-β-ocimene, with IC_50_ values of 162.3, 192.7, 193.1, 201.4, 248.6, and 273.2 µg/mL, respectively. ADMET and molecular docking modeling were the only two methods used to conduct in-depth computational analyses of compounds. The study recommended using an efficient cell suspension method to produce a volatile extract rich in useful secondary metabolites that may be utilized as a bio-insecticide.

## 1. Introduction

The various basil cultivars have the genetic ability to develop and preserve volatile compounds in different groups, culminating in a large diversity of chemical constituents in the same basil species [1,2]. Flavonoids, terpenoids, phenols, and alkaloids are some of the different types of secondary metabolites that many plants produce. Each has broad applications and biological impacts, involving anti-aging, antibacterial, and anti-inflammatory effects [3,4,5,6,7,8]. Some species of the Lamiaceae family, like *O. basilicum*, have sesquiterpenes, the phenylpropanoids’ derivatives, monoterpenes, and flavonoids in them; they could be used as bio-insecticides [9,10,11]. The basil essential oils are mostly oxygenated monoterpenes, including estragole, β-terpineol, linalool, 1,8-cineole, and nerol, following closely behind. However, they include common sesquiterpene hydrocarbons like α-guaiene, a prominent family constituent. Sesquiterpene hydrocarbons likewise comprised D, β-farnesene, β-bergamotene, and α-humulene, while monoterpene hydrocarbons were the third most important group in the extraction of *O. basilicum*, with (E)-β-ocimene being the most important. Eugenol, methyl eugenol, and chavicol were discovered to be phenylpropanoid chemicals [9,10,11].

Furthermore, *O*. *basilicum*’s essential oils have a broad range of biological properties, which could help to keep pests under control. Microorganisms have been demonstrated to be inhibited by volatile organic molecules. Furthermore, they have been widely employed in insect pest management to combat the red palm weevil (*Rhynchophorus ferrugineus*) [9], bean weevil (*Acanthoscelides obtectus*) [12], rice weevil (*Sitophilus oryzae*) [13], and cotton bollworm (*Helicoverpa armigera*) [14].

*Phoenix dactylifera* L. is a significant economic crop frequently affected by various growing pests. The worst pest to date is *Rhynchophorus ferrugineus* (Oliver). It has been discovered to infect over 21 palm species worldwide [9,15,16], resulting in agricultural output losses. Furthermore, there are several palm species that it may severely damage. The larval stage is regarded as one of the most challenging and destructive since it invades palms quickly and kills and deforms palm fronds by living on the apical meristem [17]. Through the tunnels produced by the larvae, infected palms are susceptible to infection via several insects, fungi, and pests [18]. The Mediterranean region and the Middle East, which includes Europe and North Africa, are hosts to the tropical insect *Rhynchophorus ferrugineus*, which is common and widely dispersed there. It is the most harmful for the *Rhynchophorus*’ 10 species that may be found in the pan-tropics [19,20,21,22,23].

Cells associated with plant and tissue cultures generate secondary metabolites in a controlled way in this environment. The current production and production levels are insufficient to meet the bioprocess targets for secondary metabolite synthesis via plant cells [24,25,26]. Opportunities for processes in plants are carefully examined, and new methods of producing them are developed. Plant production has been performed by utilizing somatic embryogenesis and genotype through various meristematic cell explants [9]. Therefore, somatic embryogenesis is more efficient and can be exploited to generate secondary metabolites. Prior reports have focused on optimizing plant somatic embryogenesis through modifying culture medium components involving auxins, amino acids, cytokinins, sucrose, organic additives, and basal salt formulas [25,26]. There were a few reports on plant-mediated bioactive chemical synthesis in vitro. Our previous study on the *O. basilicum*’s features as eco-insecticides versus *R. ferrugineus* is extended here. Additionally, the extracts’ growth kinetics incubated with *V. dahliae* were investigated, increasing secondary metabolite content, and identified using LC-MS and GC-MS [9,17,27,28,29,30].

This study’s hypothesis used the cell suspension procedure to generate volatile secondary metabolites, fixing a correlation between the chemicals produced by phenylpropanoids, oxygenated or hydrocarbons of sesquiterpene, and monoterpenes (secondary metabolites). Furthermore, their application as insecticides versus *R. ferrugineus* adults and larvae was investigated. The ongoing research looks into the cell suspensions’ growth dynamics to identify and synthesize secondary metabolites of *O. basilicum*. The components’ phenolic formation from cell culture was measured utilizing GC-MS (phenylpropanoids, oxygenated or hydrocarbons of sesquiterpene, and monoterpenes). The insecticidal activity of volatile secondary metabolites and the pure phenolic composition of the components were evaluated against *R. ferrugineus* larvae and adults. They were examined for their interface insecticide and antifeedant properties, as well as in vivo and in vitro inhibitory assays of the cysteine, metalloproteinases, and serine of the red palm weevil. The ADMET properties (absorption, distribution, excretion, metabolism, and toxicity) were evaluated, in addition to molecular docking, using in silico simulation. The study is anticipated to contribute to creating a natural bio-insecticide that is eco-friendly and effective against this pest.

## 2. Results

### 2.1. Cell Suspension and Callus Initiation and Total Protein Content

**Figure 1:**
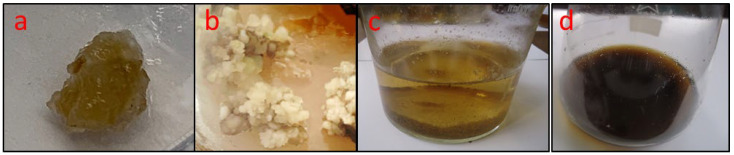
*O. basilicum* callus and cell suspension in MS and LS medium consisting of kinetin (0.5 mg/L), NAA (0.5 mg/L), 3% (*w*/*v*) sucrose, 2,4-D (0.1 mg/L), and IBA (1 mg/L); (**a**) initiation of callus after seven days, (**b**) initiation of callus after 25 days, (**c**) cell suspension after 15 days, and (**d**) cell suspension after 40 days via somatic embryogenesis. IBA: indole butyric acid; NAA: 1-naphthaleneacetic acid; and 2,4-D: 2,4-dichlorophenoxyacetic acid.

It was discovered that the callus had multiplied and been transferred to the cell suspension media by the time it was 5–6 weeks old. The LS liquid medium kept growing and produced more somatic embryos than the solid medium employed for the same purpose. The somatic embryos’ rate and amount formed and begun as greater in the cell culture when embryogenic calli were placed for 5–6 weeks for proliferation than in a solid medium (Figure 1).

The protein content in cell suspension and callus is presented in Table S1. The results from Table S1 indicated a significant elevation in protein content throughout different initiation stages of the callus or cell suspension (5–40 days) with an increase in the presence of stimulation by *V. dahliae*.

High values of protein content in *O. basilicum* presence and an absence of infection via *V. dahliae* were found at the end of the experiment (40 days). Protein content in *O. basilicum* gradually increased from 68.41 to 305.24 µg/gm in the callus without infection by *V. dahliae* and from 203.88 to 514.25 µg/gm in the callus in the case of the infection (Table S1). The data showed that the µg protein in the callus of *O. basilicum* per mL extract from the 1 g callus ratio increased with infection and the age of the callus. The µg protein/g callus ratio increased from 68.41 after five days to 305.24 at 40 days old without infection. In the infection state of *O. basilicum*, the protein content increased gradually from 203.88 to 514.25, with infection at 5 and 40 days old, respectively.

In the cell suspension, the protein content in *O. basilicum* gradually increased from 12.73 to 165.42 µg/gm cell suspension without infection by *V. dahliae*. In contrast, it was increased from 20.70 to 275.12 µg/gm cell suspension in the case of its infection. Generally, the results indicated that *V. dahliae* significantly increased the protein content in both cell suspension and callus.

### 2.2. O. basilicum Volatile Extract’s Formation and Chemical Composition

At 5–6 weeks, the callus was proliferated and then transported to the medium for cell suspension. The liquid LS medium consistently produced more somatic embryos than the solid LS medium used for the same purpose. It took around five or six weeks of proliferation in a cell suspension culture after transferring calli from embryogenic embryos, during which a greater average number of somatic embryos were generated and initiated than those gathered from the solid medium. The weights of *O. basilicum* cell suspensions produced with and without *V. dahliae* as an activator were determined.

The largest percentage of oxygenated monoterpenes was formed in the volatile methanolic extracts of *O. basilicum* (49.89%), according to GC-MS data presented in Table 1. The percentages of oxygenated monoterpenes (OMs), sesquiterpenes hydrocarbons (SHs), monoterpenes hydrocarbons (MHs), oxygenated sesquiterpenes (OSs), and phenylpropanoids in the extract were 49.89%, 21.92%, 17.46%, 4.2%, and 2%, respectively. The volatile extract’s highest component of *O. basilicum* was oxygenated monoterpenes (49.89%), which comprised 15 compounds (Table 1). Estragole accounted for oxygenated monoterpenes (22.38%) in the extract, accompanied by α-terpineol (12.37%), 1,8-cineole (7.24%), nerol (1.6%), and linalool (1.1%) (Figure S1).

Table 1 shows that there was a total of 14 components, the most abundant of which was (E)-β-ocimene (11.96%). With 4.8% of the overall volatile extract, α-guaiene was the most abundant SHs in the extract (Table 1). Germacrene D, β-farnesene, β-bergamotene, and α-humulene were presented as sesquiterpene hydrocarbons, accounting for 4.2%, 2.3%, 2.3%, and 1.52%, respectively. SHs comprised germacrene D, β-farnesene, α-humulene, and β-bergamotene in percentages of 4.2%, 2.3%, 1.52%, and 2.3%, respectively. When analyzing the extract, MHs were found to be the third largest category, with a value of 17.46%. The volatile extract was analyzed, and three phenylpropanoid chemicals—eugenol, methyl eugenol, and chavicol—were present in amounts of 3.7%, 0.4%, and 0.1%, respectively. The OSs τ-cadinol and α-eudesmol accounted for 0.1% and 1.8% of the total volatile extract (Table 1). Volatile extracts included no more than 0.7% of non-terpene derivative compounds by value (Table 1).

**Figure 2:**
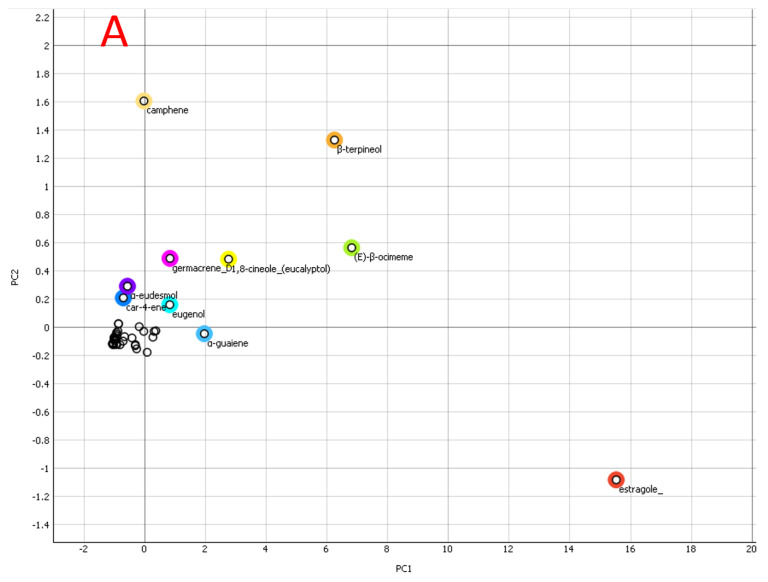

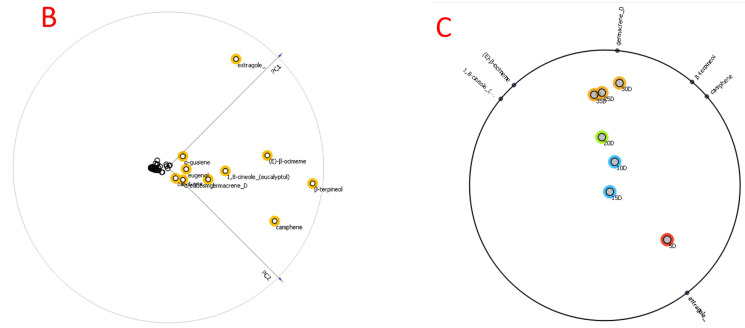
Principal component analysis (PCA) of the *O. basilicum* cell suspension extracts’ metabolite profiles throughout the initiation time (40 days); (**A**): loading plot and (**B**,**C**): score plot.

PCA was utilized to evaluate the significance between specimens and the metabolites’ involvement in the grouping. Figure 2 revealed no distinction between specimens of the same species. A big disparity between specimens suggests a featured distinction. As per the loading plot, the metabolites accountable for the greatest contribution to the cluster of 40 days (last 15 days) rapidly in cell suspension were estragole (22.38%), β-terpineol (12.37%), (E)-β-ocimene (11.96%), 1,8-cineole (7.24%), α-guaiene (4.8%), germacrene D (4.2%), eugenol (3.7%), β-bergamotene (2.3%), β-farnesene (2.3%), α-eudesmol (1.8%), nerol (1.6%), α-humulene (1.52%), linalool (1.2%), β-caryophyllene (1.1%), and γ-terpinene (1%) contributed to the separation of cell suspension after 40 days from the other samples. This means that samples after 40 days of cell suspension have a lot of estragole (22.38%), β-terpineol (12.37%), (E)-β-ocimene (11.96%), 1,8-cineole (7.24%), α-guaiene (4.8%), germacrene D (4.2%), eugenol (3.7%), β-bergamotene (2.3%), and β-farnesene (2.3%), in contrast to that of the other specimens.

**Figure 3:**
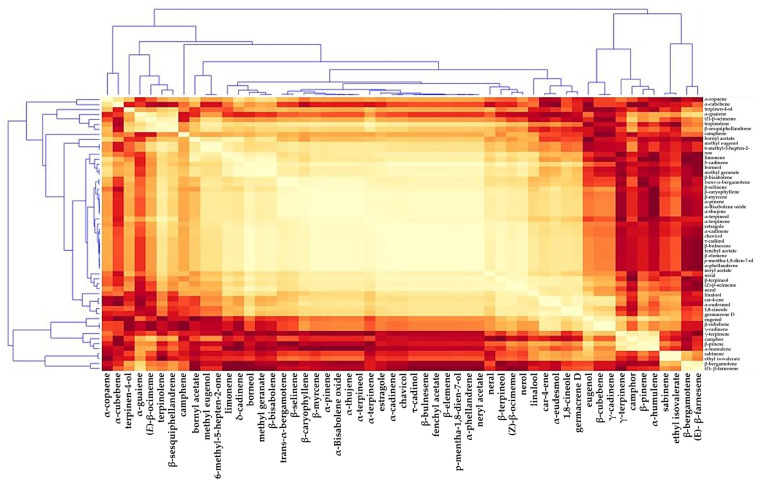
Absolute Pearson correlation heatmap distance (APCHD) and hierarchical clustering analysis (HCA) of the *O. basilicum* cell suspension extracts’ compound data throughout the initiation period (40 days). Each square represents the Pearson’s correlation coefficient of a pair of chemicals, with the correlation coefficient’s value expressed on the color scale by the intensity of yellow or orange hues.

Hierarchical clustering analysis (HCA) was conducted utilizing Pearson’s correlation and average linkage to investigate associations between the 44 metabolites’ concentrations, including extract. The data showed values for oxygenated monoterpenes (15 compounds, 49.89%), sesquiterpenes hydrocarbons (25 compounds, 21.92%), monoterpenes hydrocarbons (14 compounds, 17.46%), phenylpropanoids (3 compounds, 4.2%), and oxygenated sesquiterpenes (3 compounds, 2%). In HCA, when metabolites are placed close to each other, this demonstrates a high association (Figure 3).

In addition, the data in Figure 3 demonstrated that *O. basilicum* components elevated gradually over the first 25 days in the cell suspension. They kept elevating rapidly during the final 15 days of the test (40 days) (Figure 3).

### 2.3. Volatile Extract and Pure Components Activity against Adults and Larvae of R. ferrugineus

Table 2 demonstrates the extract’s capacity versus the adults of *R. ferrugineus*. The volatile extract was robust versus adults, with a 95% confidence limit (CL) of 1041–1392 and an LC_50_ of 1229 µg/mL.

β-Bergamotene, α-eudesmol, β-farnesene, linalool, 1,8-cineole, eugenol, α-guaiene, and β-caryophyllene had the greatest insecticidal activity with LC_50_ of 1294, 1312, 1356, 1398, 1426, 1459, 1491, and 1523 µg/mL, and 95% confidence limits of 1086–1418, 1167–1548, 1178–1572, 1176–1583, 1197–1594, 1226–1647, and 1312–1736, respectively. Adults’ moderate to low activity was expressed in nerol and germacrene D with LC_50_ of 1947 and 2045 µg/mL, 95% confidence limits of 1569–2119 and 1825–2314, respectively. (E)-β-ocimene, estragole, γ-terpinene, and β-terpineol presented low activity against adults with LC_50_ of 3014, 3587, 3841, and 3924 µg/mL, respectively.

The topical application demonstrated that the *O. basilicum*’s volatile extract LD_50_ value (µg/larva) was 13.8. α-Eudesmol, linalool, 1,8-cineole, eugenol, and nerol were shown to have the highest insecticidal activity, with LD_50_ values of 12.4, 13.7, 13.9, 14.2, and 15.6 µg/larva, respectively. β-Bergamotene, β-farnesene, α-guaiene, β-caryophyllene, and germacrene D all showed LD_50_ values on the larva, indicating moderate activity 17.2, 18.4, 19.2, 20.1, and 21.3 µg/larva, respectively. Finally, (E)-β-ocimene, estragole, γ-terpinene, and β-terpineol indicated low activity with an LD_50_ of 31.2, 34.8, 38.3, and 42.6 µg/larva, respectively.

### 2.4. O. basilicum Cell Suspension’s Extract Impact on Serine, Cysteine, and Metalloproteinase Particular Activity Assays (In Vitro)

**Figure 4:**
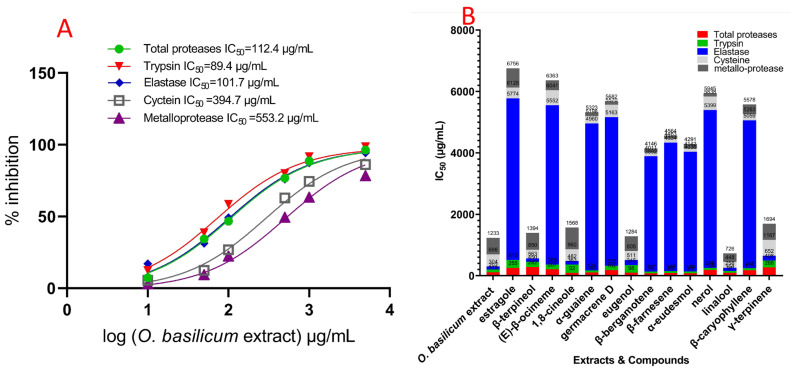
IC_50_ of the cell suspensions volatile extract of *O. basilicum*; (E)-β-ocimene, β-terpineol, estragole, 1,8-cineole, α-guaiene, germacrene D, β-bergamotene, eugenol, β-farnesene, α-eudesmol, nerol, linalool, and β-caryophyllene, and γ-terpinene towards total proteases, trypsin proteinase, chymotrypsin proteinase, elastase, cysteine protease, and metalloprotease (in vitro) of *R. ferrugineus* midgut. (**A**) Activity and IC_50_ of volatile extract versus proteases enzymes (in vitro) and (**B**) *O. basilicum* volatile extract and compounds’ IC_50_ versus proteases enzymes (in vitro) on the enzyme activity data according to one-way analysis of variance (ANOVA).

The data showed that *O. basilicum* volatile extract’s serial doses impacted IC_50_ values compared to midgut proteases from untreated larvae. Figure 4 revealed that the IC_50_ of the midgut rose with increasing levels, while the extract’s IC_50_ was 112.4 g/mL. All three elastase-like enzymes, proteinases, trypsin, and chymotrypsin, all present in midgut homogenate from 4th instar insects, are shown in Figure 4 to have varying degrees of activity and suppression. The serine proteinases’ particular activity in whole homogenate preparations is comparable and is reported as the OD/mg protein/min. The IC_50_ values were an *O. basilicum* volatile extracts’ significant impact. Trypsin proteinases and elastase have much higher IC_50_ values for suppression by *O. basilicum* volatile extract in midgut homogenate preparations than chymotrypsin-like serine proteinase. The IC_50_ value for the volatile extract is displayed in Figure 4 (in vitro). This demonstrated that suppression differed considerably among midgut homogenates. Both trypsin and elastase proteinase activities were rather high in the fourth instar midgut sample (4.14 and 1.28, respectively). Likewise, the *O. basilicum*’s active volatile extract had an IC_50_ of 89.4 and 101.7 µg/mL, respectively. These extracts can potentially inhibit trypsin proteinases originating from the fourth midgut. Metalloprotease and cysteine both have a significant inhibitory reactivity to volatile extract; Figure 4 displays the IC_50_ values, which showed that the volatile extract had inhibitory activity at IC_50_ concentrations of 535.2 and 394.7 µg/mL, respectively. Chymotrypsin proteinases behaved differently than serine proteinases; the IC_50_ values (Figure 4) indicated that the volatile extract did not suppress chymotrypsin when the IC_50_ value was greater than 5000 µg/mL. In Figure 4, we see the effect of the individual components on the total protease activity measured in the *R. ferrugineus* larval midgut during its fourth instar. Different dosages significantly affected the IC_50_ rates for all examined substances compared to untreated larvae. Figure 4 depicts the influence of compounds on the *R. ferrugineus* fourth instar larval midgut’s overall protease activity (in vitro). Significant influence was exerted by the varying IC_50_ values of all substances examined.

Β-Bergamotene, α-eudesmol, β-farnesene, linalool, 1,8-cineole, eugenol, α-guaiene, and β-caryophyllene were expressed activity versus total proteases, with IC_50_ values of 78.9, 81.2, 88.6, 90.7, 91.5, 97.6, 107.4, and 176.3 µg/mL, respectively. Germacrene D, nerol, (E)-β-ocimene, estragole, γ-terpinene, and β-terpineol expressed moderated activity versus total proteases, with IC_50_ values of 182.1, 188.3, 207.4, 254.7, 268.1, and 281.7 µg/mL, respectively. β-Bergamotene, α-eudesmol, β-farnesene, linalool, α-guaiene, nerol, and β-caryophyllene indicated more significant impact versus trypsin, with IC_50_ values of 58.5, 58.9, 60.2, 63.4, 66.8, 68.1, and 70.4 µg/mL, respectively. Germacrene (E)-β-ocimene, β-terpineol, γ-terpinene, eugenol, estragole, and 1,8-cineole expressed moderated activity versus trypsin proteases, with IC_50_ values of 144.2, 157.2, 168.4, 229.7, 247.2, 257.5, and 271.2 µg/mL, respectively. Linalool, 1,8-cineole, β-terpineol, eugenol, and γ-terpinene inhibited elastase activity with IC_50_ values of 104.2, 119.4, 112.6, 165.7, and 154.3 µg/mL, respectively. Moreover, the inhibitory impact of reminding substances (IC_50_ > 3750 µg/mL) was not observed. Figure 4 clarifies the IC_50_ values for chymotrypsin, indicating that no compounds had an inhibitory impact with IC_50_ > 5000 µg/mL. Metalloproteases, β-farnesene, nerol, germacrene D, α-guaiene, β-bergamotene, and α-eudesmol caused a highly specific and potent inhibition response, with IC_50_ values of 101.5, 106.7, 112.3, 124.9, 134.8, and 149.4 µg/mL, respectively, showing that these substances exerted their highest inhibition at concentrations below 150 µg/mL (Figure 4). Linalool, β-caryophyllene, (E)-β-ocimene, eugenol, γ-terpinene, β-terpineol, estragole, and 1,8-cineole expressed low activity versus metalloproteases, with IC_50_ values of 278.2, 314.5, 321.7, 478.6, 526.8, 543.5, 627.8, and 708.2 µg/mL, respectively. Furthermore, cysteine showed an inhibition response to α-eudesmol, β-bergamotene, and β-farnesene compounds, which expressed the highest inhibition with values of 105.2, 119.6, and 128.4 µg/mL, respectively (IC_50_ ˂ 150 µg/mL). Linalool, β-caryophyllene, α-guaiene, β-terpineol, and eugenol were less in their IC_50_ value, with 189.2, 204.4, 238.5, 287.3, and 294.4 µg/mL, respectively.

When protease inhibitors were used, they were involved in examining protease activity to identify the presence of numerous gut proteases in the instar of the fourth larval period. As depicted in Figure S2, inhibitors drastically decreased extracted gut proteases from the fourth larval instar. Furthermore, the inhibitors of chymotrypsin proteinase TPCK, trypsin proteinase TLCK, the cysteine protease inhibitor, iodoacetic acid, metalloprotease inhibitors, 1,10 phenanthroline, EDTA, and EGTA significantly inhibited the midgut instar development (Figure S2).

### 2.5. In Vivo Effect of O. basilicum Volatile Extract and Pure Components on the Serine, Metalloprotease, and Cysteine Protease Activities from Fourth R. ferrugineus Instar Midgut Preparations 

**Figure 5:**
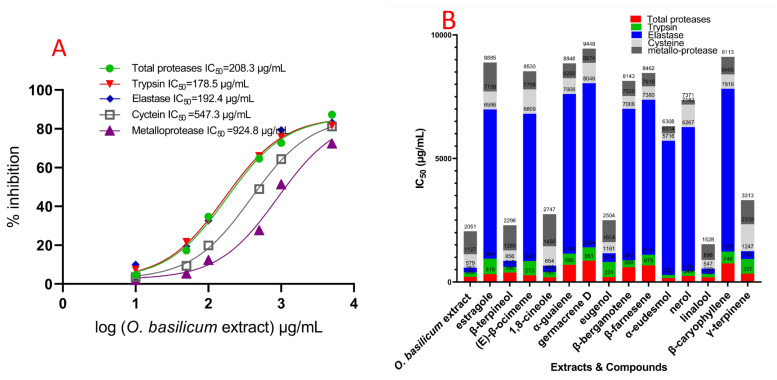
IC_50_ of the cell suspensions volatile extract of *O. basilicum*; (E)-β-ocimene, β-terpineol, estragole, 1,8-cineole, α-guaiene, germacrene D, β-bergamotene, eugenol, β-farnesene, α-eudesmol, nerol, linalool, β-caryophyllene, and γ-terpinene towards total proteases, trypsin proteinase, chymotrypsin proteinase, elastase, cysteine protease, and metalloprotease (in vivo) of *R. ferrugineus* midgut. (**A**) Activity and volatile extract’s IC_50_ versus proteases enzymes (in vivo) and (**B**) IC_50_ of *O. basilicum* volatile extract and compounds versus proteases enzymes (in vivo) on the enzyme activity data according to one-way analysis of variance (ANOVA).

The *R. ferrugineus*’ IC_50_ value’s fourth instar larvae midgut was elevated progressively with *O. basilicum* volatile extract’s concentrations (Figure 5). The *O. basilicum* volatile extract demonstrated a significant impact as per the IC_50_ value. The preparations made from the midgut of a fourth-instar larva showed an activity of 1.98 OD/mg protein/min.

In the fourth midgut preparation, the volatile extract inhibited the trypsin proteinase, elastase proteinases, metalloprotease, and cysteine proteases to a larger extent than in vitro values. Activity versus trypsin proteinase, total proteases, and elastase was observed at IC_50_ values of 178.5, 208.3, and 192.4 µg/mL, respectively, for the volatile extract of *O. basilicum*. The inhibition impact of cysteine and metalloprotease demonstrated that the volatile extract has the greatest inhibitory impact with values of 547.3 and 924.8 µg/mL, respectively (Figure 5). Figure 5 indicates the compound’s impact on overall in vivo protease activities.

The α-eudesmol, linalool, 1,8-cineole, eugenol, nerol, and (E)-β-ocimene showed activity versus overall proteases, achieving IC_50_ values of 162.3, 192.7, 193.1, 201.4, 248.6, and 273.2 µg/mL, respectively. Estragole, γ-terpinene, and β-terpineol exhibited moderate activity against total protease, with IC_50_ values of 314.8, 337.4, and 379.5 µg/mL, respectively. β-Bergamotene, β-farnesene, α-guaiene, β-caryophyllene, and germacrene D activity versus overall protease demonstrated the lowest inhibition, with IC_50_ values of 598.3, 675.2, 694.7, 748.2, and 861.2 µg/mL, respectively. α-Eudesmol, linalool, nerol, (E)-β-ocimene, and β-terpineol had a more substantial impact versus trypsin, with IC_50_ values of 124.2, 137.5, 186.3, 214.8, and 237.1 µg/mL, respectively. In addition, β-bergamotene, β-farnesene, α-guaiene, and β-caryophyllene had a non-significant impact versus trypsin, with IC_50_ values of 284.5, 425.8, 454.4, and 489.3 µg/mL, respectively. β-Caryophyllene, (E)-β-ocimene, γ-terpinene, eugenol, and estragole activity versus trypsin demonstrated the lowest inhibition with IC_50_ values of 542.8, 572.3, 594.1, 612.8, and 628.7 µg/mL, respectively. The elastase action revealed inhibitory impacts, with IC_50_ values of 239.4, 256.3, 315.8, 217.2, and 346.8 µg/mL for β-terpineol, 1,8-cineole, γ-terpinene, Linalool, and eugenol, respectively. The remaining studied chemicals had insignificant inhibition, with IC_50_ > 5000 µg/mL. Metalloproteases’ response to nerol and α-eudesmol inhibition exhibited IC_50_ values of 189.1 and 294.2 µg/mL, respectively. Moreover, β-farnesene, germacrene D, α-guaiene, and β-bergamotene indicated less action versus metalloproteases having IC_50_ ˃ 300 µg/mL, and less than 1000 µg/mL with values of 545.7, 574.6, 594.7, 613.5, 632.4, 708.2, 735.6, 889.4, and 974.2 µg/mL, respectively. Estragole, β-terpineol, and 1,8-cineole indicated the lowest action versus metalloproteases having IC_50_ ˃ 10,000 µg/mL, with values of 1014.1, 1175.2, and 1297.2 µg/mL, respectively. Furthermore, cysteine proteases demonstrated an inhibitory reaction to α-eudesmol, linalool, β-terpineol, and eugenol (Figure 5) with IC_50_ ˂ 500 µg/mL with values of 298.3, 348.2, 427.4, and 453.1 µg/mL, respectively. Additionally, β-bergamotene, β-farnesene, β-caryophyllene, and α-guaiene demonstrated less activity with IC_50_ values of 521.4, 536.8, 586.3, and 642.3 µg/mL, respectively. Figure 5 displays the IC_50_ values of chymotrypsin, indicating that the extract had no inhibitory effect on chymotrypsin when IC_50_ > 5000 µg/mL. The same pattern was observed in metalloprotease, serine proteinase, and cysteine protease. Specific activity was not observed in chymotrypsin-like serine proteinases.

### 2.6. Docking of Compounds into Proteinase Enzymes

#### 2.6.1. Serine Proteinase Docking

**Figure 6:**
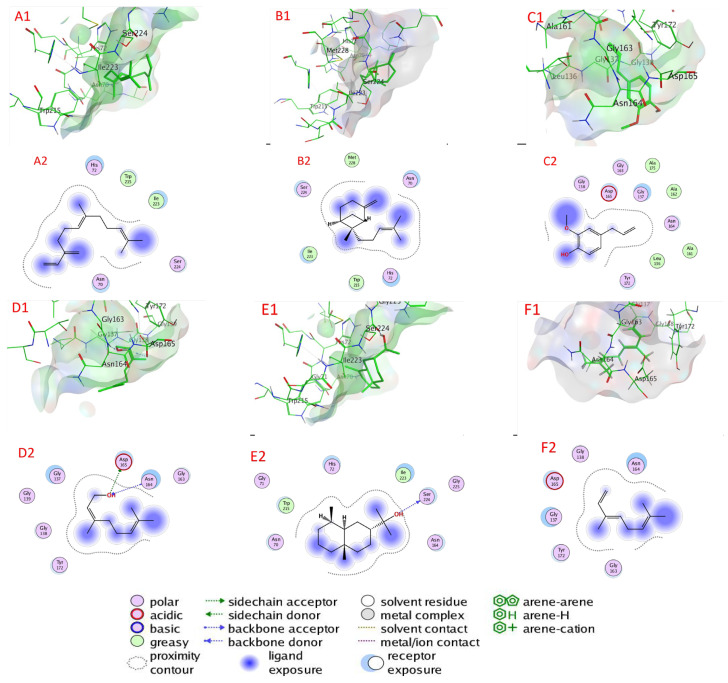
Docking view; (**A1**,**A2**) β-farnesene, (**B1**,**B2**) β-bergamotene, (**C1**,**C2**) eugenol, (**D1**,**D2**) nerol, (**E1**,**E2**) α-eudesmol, and (**F1**,**F2**) (E)-β-ocimene in the binding sites of serine proteinase (PDB:3F7O). (**A1**,**B1**,**C1**,**D1**,**E1**,**F1**) 3D complex structures (stereoview) and (**A2**,**B2**,**C2**,**D2**,**E2**,**F2**) 2D interaction diagram structures.

Table 3, Figure 6, and Figure S3 illustrate the chemicals’ docking ratings to serine proteinase (PDB:3F7O). It was discovered, based on the findings of the docking investigation, that the compounds under research had low energy for docking, ranging from −4.0485 (1,8-cineole) to −5.0728 (β-farnesene) kcal/mol, and a strong affinity again for the binding sites (target) of the serine protease (Table 3). β-Farnesene, β-bergamotene, eugenol, nerol, α-eudesmol, and (E)-β-ocimene had a higher affinity for binding than molecules with lower docking energies, with values of −5.0728, −4.9929, −4.9100, −4.7396, −4.6450, and −4.5195 kcal/mol, respectively, accompanied by α-guaiene, β-caryophyllene, germacrene D, estragole, β-terpineol, γ-terpinene, linalool, and 1,8-cineole with docking energies of −4.4206, −4.4190, −4.4132, −4.3709, −4.1040, −4.1591, −4.0872, and −4.0485 kcal/mol, respectively. On the other hand, with −4.0485 kcal/mol docking energy, 1,8-cineole had the poorest serine protease binding ability (Table 3).

β-Farnesene bonded to amino acids Asn 70, His 72, Trp 215, Gly Ile 223, and Ser 224 through van der Waals interactions as serine proteinase’s active sites. Likewise, β-bergamotene bonded to amino acids Asn 70, His 72, Trp 215, Gly Ile 223, Ser 224, and Met 228 through van der Waals interactions. Eugenol bonded to amino acids Leu 136, Gly 137, Gly 138, Ala 161, Ala 162, Gly 163, Asn 164, Asp 165, Tyr 172, and Ala 172 through van der Waals interactions. However, nerol attached via H–bonds with Asp 165 (3.19 Å), Asp 165 (2.87 Å), Asn 164 (3.08 Å), and through van der Waals bonds to Gly 137, Gly 138, Gly 139, Gly 163, and Tyr 172. Furthermore, α-eudesmol attached via H– bonds with Ser 224 (2.83 Å) and through van der Waals bonds to amino acids Asn 70, Gly 70, His 72, Asn 164, Trp 215, Ile 223, Ser 224, and Gly 225. In addition, (E)-β-ocimene bonded to amino acids Leu 136, Gly 137, Gly 138, Gly 163, Asn 164, Asp 165, and Tyr 172 through van der Waals bonds.

#### 2.6.2. Cysteine Protease Docking

**Figure 7:**
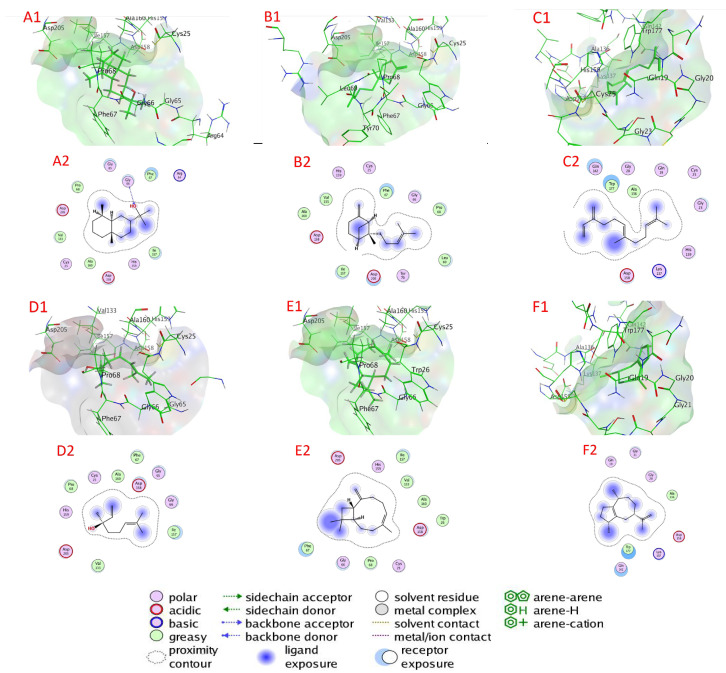
Docking view; (**A1**,**A2**) α-eudesmol, (**B1**,**B2**) β-bergamotene, (**C1**,**C2**) β-farnesene, (**D1**,**D2**) linalool, (**E1**,**E2**) β-caryophyllene, and (**F1**,**F2**) α-guaiene in the binding sites of cysteine protease (PDB:3IOQ). (**A1**,**B1**,**C1**,**D1**,**E1**,**F1**) 3D complex structures (stereoview) and (**A2**,**B2**,**C2**,**D2**,**E2**,**F2**) 2D interaction diagram structures.

Table 3 and Figure 7 and Figure S4 illustrate the chemicals’ docking ratings to cysteine protease (PDB:3IOQ). It was discovered, based on the findings of the docking investigation, that the compounds under research had low energy for docking, ranging from −4.1835 (γ-terpinene) to −5.2551 (α-eudesmol) kcal/mol, and a strong affinity for the binding sites (target) of cysteine protease (Table 3). α-Eudesmol, β-bergamotene, β-Farnesene, linalool, β-caryophyllene, and α-guaiene have a higher affinity for binding than molecules with lower docking energies, with values of −5.2551, −5.0760, −5.0141, 4.9738, −4.8315, and −4.8005 kcal/mol, respectively, accompanied by β-terpineol, eugenol, estragole, 1,8-cineole, germacrene D, nerol, (E)-β-ocimene, and γ-terpinene with docking energies of −4.6980, −4.6507, −4.5151, −4.4759, −4.4563, −4.4430, −4.4243, and −4.1835 kcal/mol, respectively. Conversely, with −4.1835 kcal/mol docking energy, γ-terpinene had the poorest cysteine protease binding ability (Table 3).

α-Eudesmol attached through H–bonds with Gly 66 (2.85 Å) and attached through van der Waals bonds to amino acids Cys 25, Arg 64, Gly 65, Phe 67, Pro 68, Val 133, Ile 157, Asp 158, His 159, Ala 160, and Asp 205 as active sites of cysteine protease. β-Bergamotene bonded to amino acids Cys 25, Gly 66, Phe 67, Pro 68, Leu 69, Tyr 70, Val 133, Ile 157, Asp 158, His 159, Ala 160, and Asp 205 through van der Waals interactions. Additionally, β-farnesene is bonded to amino acids Gln 19, Gly 20, Gly 23, Cys 25, Ala 136, Lys 137, Gln 142, Asp 158, His 159, and Trp 177 through van der Waals bonds. Linalool bonded to amino acids Cys 25, Gly 65, Gly 66, Phe 67, Pro 68, Val 133, Ile 157, Asp 158, His 159, Ala 160, and Asp 205 through van der Waals interactions. Additionally, β-caryophyllene bonded to amino acids Cys 25, Trp 26, Gly 66, Phe 67, Pro 68, Val 133, Ile 157, Asp 158, His 159, Ala 160, and Asp 205 through van der Waals bonds. α-Guaiene bonded to amino acids Gln 19, Gly 20, Gly 21, Ala 136, Lys 137, Gln 142, Asp 158, and Trp 177 through van der Waals interactions.

#### 2.6.3. Metalloprotease Docking

**Figure 8:**
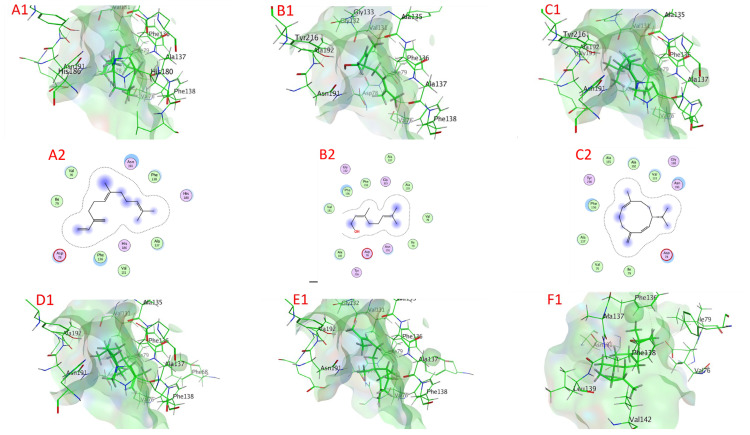

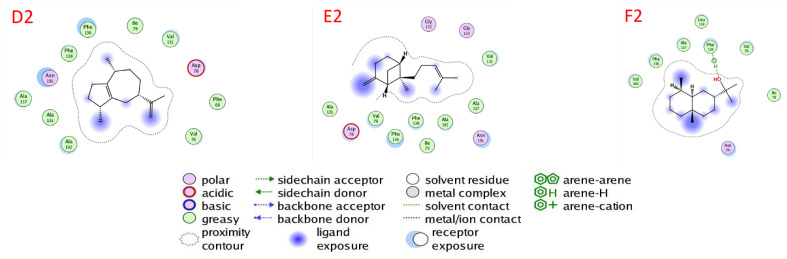
Docking view: (**A1**,**A2**) β-farnesene, (**B1**,**B2**) nerol, (**C1**,**C2**) germacrene D, (**D1**,**D2**) α-guaiene, (**E1**,**E2**) β-bergamotene, and (**F1**,**F2**) α-eudesmol in the binding sites of metalloprotease (PDB:1KAP). (**A1**,**B1**,**C1**,**D1**,**E1**,**F1**) 3D complex structures (stereoview) and (**A2**,**B2**,**C2**,**D2**,**E2**,**F2**) 2D interaction diagram structures.

Table 3 and Figure 8 and Figure S5 illustrate the chemicals’ docking ratings to metalloprotease (PDB:1KAP). It was discovered, based on the findings of the docking investigation, that the compounds under research had a low docking energy, ranging from −4.2706 (1,8-cineole) to −5.6725 (β-farnesene) kcal/mol, and a strong affinity for the binding sites (target) of metalloprotease (Table 3). β-Farnesene, nerol, germacrene D, α-guaiene, β-bergamotene, and α-eudesmol had a higher affinity for binding than molecules with lower docking energies, with values of −5.6725, −5.5082, −5.4350, −5.3298, −5.3167, and −5.2549 kcal/mol, respectively, accompanied by linalool, β-caryophyllene, (E)-β-ocimene, eugenol, γ-terpinene, estragole, 1,8-cineole, and β-terpineol with docking energies of −5.0587, −5.0288, −4.9996, −4.8218, −4.7142, −4.6842, −4.2706, and −4.6411 kcal/mol, respectively. On the other hand, with −4.2706 kcal/mol docking energy, 1,8-cineole had the poorest metalloprotease binding ability (Table 3).

β-Farnesene bonded to amino acids Val 76, Asp 78, Ile 79, Val 131, Phe 136, Ala 137, Phe 138, His 180, His 186, and Asn 191 through van der Waals interactions as active sites of metalloprotease. Nerol bonded to amino acids Val 76, Asp 78, Ile 79, Val 131, Gly 132, Gly 133, Ala 135, Phe 136, Ala 137, Phe 138, Asn 191, Ala 192, and Tyr 216 through van der Waals bonds. In addition, germacrene D bonded to amino acids Val 76, Asp 78, Ile 79, Val 131, Ala 135, Phe 136, Ala 137, Asn 191, Ala 192, Gly 193, and Tyr 216 through van der Waals interactions. α-Guaiene bonded to amino acids Phe 68, Val 76, Asp 78, Ile 79, Val 131, Ala 135, Phe 136, Ala 137, Phe 138, Asn 191, and Ala 192 through van der Waals interactions. Additionally, β-bergamotene bonded to amino acids Val 76, Asp 78, Ile 79, Val 131, Gly 132, Gly 133, Ala 135, Phe 136, Ala 137, Phe 138, Asn 191, and Ala 192 through van der Waals interactions. However, α-eudesmol connected via H-pi bond with Phe 138 (3.93 Å) and bonded to amino acids Val 76, Ile 79, Phe 136, Ala 137, Leu 139, Val 142, and Asn 191 through van der Waals interactions.

#### 2.6.4. Trypsin Proteinase Docking

**Figure 9:**
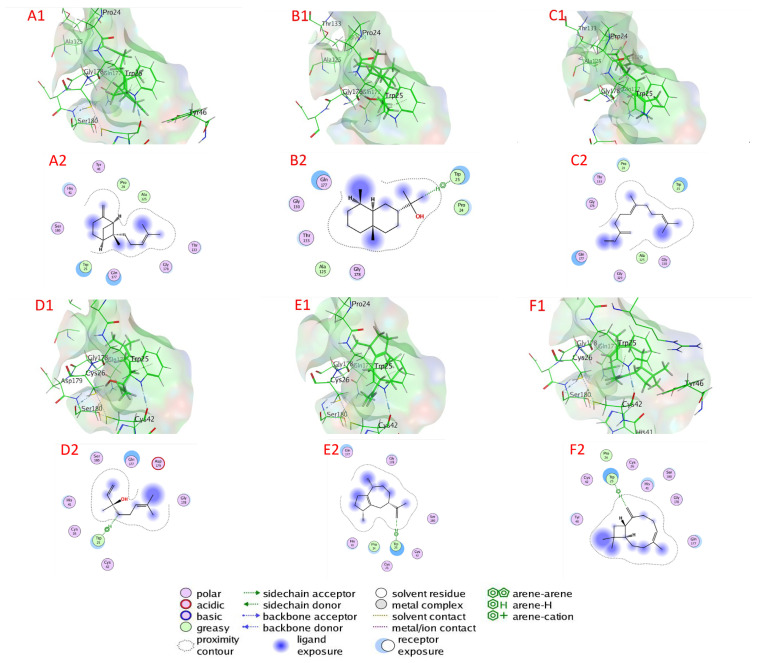
Docking view; (**A1**,**A2**) β-bergamotene, (**B1**,**B2**) α-eudesmol, (**C1**,**C2**) β-farnesene, (**D1**,**D2**) linalool, (**E1**,**E2**) α-guaiene, and **(F1**,**F2**) β-caryophyllene in the binding sites of trypsin proteinase (PDB:1FN8). (**A1**,**B1**,**C1**,**D1**,**E1**,**F1**) 3D complex structures (stereoview) and (**A2**,**B2**,**C2**,**D2**,**E2**,**F2**) 2D interaction diagram structures.

Table 3 and Figure 9 and Figure S6 illustrate the chemicals’ docking ratings to trypsin proteinase (PDB:1FN8). It was discovered, based on the findings of the docking investigation, that the compounds under research had a low docking energy, ranging from −4.2897 (1,8-cineole) to −5.2044 (β-Bergamotene) kcal/mol, and a strong affinity for the binding sites (target) of trypsin proteinase (Table 3). β-Bergamotene, α-eudesmol, β-farnesene, linalool, α-guaiene, and β-caryophyllene has a higher affinity for binding than molecules with lower docking energies, with values of −5.2044, −5.1964, −5.1448, −4.9127, 4.8352, and −4.7619 kcal/mol, respectively, accompanied by nerol, germacrene D, (E)-β-ocimene, β-terpineol, γ-terpinene, eugenol, estragole, 1,8-cineole with docking energies of −4.7201, −4.5790, −4.5755, −4.4.4555, −4.4256, −4.3781, −4.3164, and −4.2897 kcal/mol, respectively. On the other hand, with −4.2897 kcal/mol docking energy, 1,8-cineole had the poorest trypsin proteinase binding ability (Table 3).

β-Bergamotene bonded to amino acids Pro 24, Trp 25, His 41, Tyr 46, Ala 125, Thr 133, Gln 177, Gly 178, and Ser 180 through van der Waals interactions as active sites of trypsin proteinase. However, α-eudesmol connected via H–pi bonds with Trp 25 (3.64 Å) and bonded to amino acids Ala 125, Gly 130, Thr 133, Gln 177, and Gly 178 through van der Waals interactions. β-Farnesene bonded to amino acids Pro 24, Trp 24, Ala 125, Gly 129, Gly 130, Thr 133, Gln 177, and Gly 178 through van der Waals interactions. In addition, linalool connected via H–pi bonds with 5-ring Trp 25 (4.54 Å) and 6-ring Trp 25 (3.77 Å) while attached through van der Waals interactions with amino acids Cys 26, His 41, Cys 42, Gln 177, Gly 178, Asp 179, and Ser 180. α-Guaiene is connected via H-pi bonds with 5-ring Trp 25 (3.83 Å) and attached through van der Waals interactions with amino acids Pro 24, Cys 26, His 41, Cys 42, Gln 177, Gly 178, and Ser 180. Additionally, β-caryophyllene connected via H-pi bonds with 5-ring Trp 25 (4.13 Å) and bonded to amino acids Pro 24, Cys 26, His 41, Cys 42, Try 46, Gln 177, Gly 178, and Ser 180 through van der Waals interactions.

### 2.7. ADMET Analysis

Table S1 outlines the evaluation of various descriptors involving HBA, BBB, PPB, HBD, LogS, LogP, H-HT, and CYP450 of compounds depicted in Table S1. Table S2 depicts estragole, 1,8-cineole, eugenol, nerol, β-terpineol, and linalool with LogP values ranging from 2.129 to 2.744. At the same time, (E)-β-ocimene, α-guaiene, β-bergamotene, germacrene D, β-farnesene, α-eudesmol, and α-humulene had LogP values ˃3. LogS values of estragole, 1,8-cineole, β-terpineol, nerol, eugenol, and linalool were more than 60 μg/mL, while for (E)-β-ocimene, α-eudesmol, and γ-terpinene LogS values were 10–60 μg/mL. Moreover, α-guaiene, β-bergamotene, β-farnesene, germacrene D, and α-humulene had LogS values ˂10 μg/mL. The values of LogS and LogP that obtained from α-guaiene, β-bergamotene, germacrene D, β-farnesene, and α-humulene equal 0.267, 0.259, 0.175, 0.11, and 0.062 μg/mL, and 4.725, 4.891, 4.725, 5.202, and 5.035, respectively (Table S2).

In addition, estragole, 1,8-cineole, β-terpineol, eugenol, α-eudesmol, nerol, and linalool indicated a raised polar surface area (PSA), revealing an inability to penetrate cell membranes as presented in PSA values of 9.23, 20.23, 9.23, 29.46, 20.23, 20.23, and 20.23, respectively. At the same time, (E)-β-ocimene, α-guaiene, β-bergamotene, germacrene D, α-humulene, and β-farnesene demonstrated nonpolar surface area and cell membrane penetration ability with PSA values equal to zero. Moreover, CYP450 had a negative association with β-terpineol, 1,8-cineole, α-guaiene, germacrene D, β-bergamotene, α-eudesmol, nerol, α-humulene, linalool, β-caryophyllene, and γ-terpinene, while having a positive association with estragole (+++), (E)-β-ocimene (+), eugenol (+++), β-farnesene (+), and γ-terpinene (+). PPB and the hepatotoxicity (H-HT) for all compounds revealed increasing positive and negative values with all compounds.

## 3. Discussion

The present study investigated the volatile secondary metabolites from *O. basilicum*’s cell suspension extract. β-Bergamotene, α-eudesmol, β-farnesene, linalool, 1,8-cineole, eugenol, α-guaiene, and β-caryophyllene had a significant insecticidal action versus *R. ferrugineus*’ adults. α-Eudesmol, linalool, 1,8-cineole, eugenol, and nerol revealed the greatest objective application activities versus larvae. The research reported that volatile extracts were effective versus overall proteases (in vitro and in vivo) and particular activities versus trypsin proteases and elastases. In cell suspensions obtained from *O. basilicum*, especially after *V. dahliae* activator inoculation, the synthesis of secondary metabolites such as oxygenated sesquiterpenes, oxygenated monoterpenes, oxygenated sesquiterpenes, monoterpene hydrocarbons, and phenylpropanoids compounds increased rapidly. It was established that the volatile extract from *O. basilicum* effectively and efficiently improved deterrence and decreased *R. ferrugineus* larvae feeding. However, the antifeedant impact is strongest in adults.

Moreover, according to our data, the overall rate of antifeedant activities was observed and discovered in optimal circumstances. The volatile extract demonstrated substantial insecticidal action, which may be due to the bioactive metabolites’ range present in the extract. *O. basilicum* oxygenated monoterpenes, sesquiterpene hydrocarbons, oxygenated sesquiterpenes, and monoterpene hydrocarbons from cell suspension raised progressively during the first 25 days of the test, then quickly accelerated and kept growing quickly for the final fifteen days. For the substances tested using the IC_50_ rate, various dosages had a significant impact. Comparing the compounds’ influence against midgut protease activities (in vitro) generated by fourth-stage *R. ferrugineus*, larvae’s midgut revealed the effective action of *O. basilicum*’s volatile extract.

The volatile extract’s LC_50_ in adults was 1229 µg/mL. The LC_50_ values of β-bergamotene, α-eudesmol, β-farnesene, linalool, 1,8-cineole, eugenol, α-guaiene, and β-caryophyllene had the greatest insecticidal activity with values of 1294, 1312, 1356, 1398, 1426, 1459, 1491, and 1523 µg/mL, respectively. Medium-to-low levels of activities in adults were expressed in nerol and germacrene D with LC_50_ of 1947 and 2045 µg/mL, respectively.

The volatile extract had an LD_50_ of 13.8 (µg/larva). α-Eudesmol, linalool, 1,8-cineole, eugenol, and nerol revealed the greatest insecticidal activity with LD_50_ values of 12.4, 13.7, 13.9, 14.2, and 15.6 µg/larva, respectively. (E)-β-ocimene, estragole, γ-terpinene, and β-terpineol showed low activity with LD_50_ values of 31.2, 34.8, 38.3, and 42.6 µg/larva, respectively. (E)-β-Ocimene, estragole, γ-terpinene, and β-terpineol presented low activity against adults with LC_50_ values of 3014, 3587, 3841, and 3924 µg/mL, respectively.

The basil’s active volatile extract revealed inhibitory activities versus trypsin proteinases, elastase, total protease, cysteine, and metalloprotease activity with IC_50_ values of 89.4, 101.7, 112.4, 394.7, and 535.2 µg/mL, respectively. Nevertheless, the *O. basilicum* volatile extracts’ action versus proteases enzymes indicated an association with β-bergamotene (2.3%), α-eudesmol (1.8%), β-farnesene (2.3%), linalool (1.2%), 1,8-cineole (7.24%), eugenol (3.7%), α-guaiene (4.8%), and β-caryophyllene (1.1%) contents.

β-Bergamotene, α-eudesmol, β-farnesene, linalool, 1,8-cineole, eugenol, α-guaiene, and β-caryophyllene had inhibition impacts versus total proteases, with IC_50_ values of 78.9, 81.2, 88.6, 90.7, 91.5, 97.6, 107.4, and 176.3 µg/mL, respectively. The extract’s action versus trypsin is linked with β-bergamotene, α-eudesmol, β-farnesene, linalool, α-guaiene, nerol, and β-caryophyllene with IC_50_ values of 58.5, 58.9, 60.2, 63.4, 66.8, 68.1, and 70.4 µg/mL (2.3%, 1.8%, 2.3%, 1.2%, 4.8%, 1.6%, and 1.1% contents), respectively. The inhibitory activities of linalool, β-terpineol, 1,8-cineole, eugenol, and γ-terpinene versus elastase activities were revealed by IC_50_ values of 104.2, 112.6, 119.4, 165.7, and 154.3 µg/mL (7.24%, 1%, 3.7%, 12.37%, and 1.2% contents), respectively. β-Farnesene, nerol, germacrene D, α-guaiene, β-bergamotene, and α-eudesmol had the greatest responsibility of inhibition impact versus metalloproteases with IC_50_ ˂ 150 µg/mL, and values of 101.5, 106.7, 112.3, 124.9, 134.8, and 149.4 µg/mL, (1.2%, 1.6%, 4.2%, 4.8%, 2.3%, and 1.8% contents), respectively. On the contrary, α-eudesmol, β-bergamotene, and β-farnesene presented specific inhibitory impacts versus cysteine where IC_50_ < 150 µg/mL was based on the values of 105.2, 119.6, and 128.4 µg/mL, (1.8%, 2.3%, and 2.3% contents), respectively. These data confirm that chemicals affect protease activity (in vitro and in vivo), including α-eudesmol, linalool, 1,8-cineole, eugenol, nerol, and (E)-β-ocimene, having activity versus overall proteases. It can be deduced that α-eudesmol, linalool, 1,8-cineole, eugenol, nerol, and (E)-β-ocimene impacted insecticidal activity versus *R. ferrugineus*’ proteases enzymes.

In addition, as these volatile extracts are content antifeedant compounds, site targets and mechanisms of action in these insects have been unknown until now. Based on the research [31], insecticidal action pathways may entail changing feeding physiology, long-term toxicity, or repellency.

Importantly, these secondary molecules are acknowledged as important components once they develop in cells and play a critical role in pathogen defense [32,33,34,35]. PGR is needed to control how plants develop and differentiate, which could control how anabolism destroys phenolic contents [36,37]. Therefore, cutting-edge methods like bioactive secondary metabolite green biosynthesis are highly sought after now [38,39,40]. Furthermore, due to its short response time, ability to divide cells, and ease of use, cell suspension culture is a quicker and more efficient method than callus culture for boosting the production of bioactive chemicals [9,26,40,41].

The difference in the *O. basilicum* volatile extracts’ secondary metabolites across in vitro and in vivo protease inhibition tests (Figure 4 and Figure 5) clarified proteinases’ vital role in their mechanism [20,42]. The results demonstrated that toxicity might present an outcome of α-eudesmol (1.8%), linalool (1.2%), 1,8-cineole (7.24%), eugenol (3.7%), nerol (1.6%), and (E)-β-ocimene (11.96%) in the volatile extract of *O. basilicum* and possibly towards the importance of proteinases.

The suppression of trypsin proteinase, cysteine protease, metalloprotease, and elastase proteinases via the *O. basilicum*’s volatile extract was definitively proven fourth midgut levels were lower than in vitro values. Numerous in vivo values exhibit the equivalent pattern and are less than their in vitro counterparts. The in vivo test of β-farnesene, β-bergamotene, α-guaiene, and β-caryophyllene activity versus overall protease revealed that compounds suppress overall protease the least in vitro.

Estragole, β-terpineol, (E)-β-ocimene, 1,8-cineole, eugenol, α-eudesmol, nerol, linalool, and γ-terpinene are exceedingly hydrophobic, allowing them to pass through biological membranes, as determined by the ADMET screening study. Moreover, α-guaiene, β-bergamotene, β-farnesene, germacrene D, and β-caryophyllene have poor aqueous solubility with LogP values of 4.425, 4.725, 5.202, 4.891, and 4.725 and low solubility with LogS values of 0.269, 0.259, 0.175, 0.11, and 0.275 log mol/L, respectively. Dependent on these results, the Lipinski rules showed that eugenol, β-terpineol, 1,8-cineole, linalool, nerol, estragole, α-eudesmol, γ-terpinene, and (E)-β-ocimene had a great theoretical bioavailability orally. The chemicals’ effectiveness (α-eudesmol, linalool, 1,8-cineole, eugenol, and nerol) as bio-insecticides was assessed based on their potency against the target insect and their ADMET profiles. When it comes to metabolic aspects, β-terpineol, 1,8-cineole, α-guaiene, germacrene D, β-bergamotene, α-eudesmol, nerol, α-humulene, linalool, β-caryophyllene, and γ-terpinene were incapable of inhibiting CYP450 enzymes, suggesting their metabolic durability versus CYP450 enzymes. In contrast, estragole (+++), eugenol (+++), (E)-β-ocimene (+), β-farnesene (+), and γ-terpinene (+) exhibited inhibition versus CYP450 enzymes. Nevertheless, the results of hepatotoxicity anticipation recommended that no compounds had any toxic impact on liver cells.

Docking research revealed that investigated ligands create ionic and metallic interactions with van der Waals interactions and hydrogen bonding between amino acids, directing the molecule to the active sites of enzymes, including serine proteinases, cysteine proteinases, elastase, and metalloproteases [26,43]. The greatest docking binding to serine proteinase and metalloprotease was displayed by β-farnesene, ΔG = −5.0728, and ΔG = −5.6725, respectively. The α-eudesmol’s binding confirmation offered the greatest docking (ΔG = −5.2551) with cysteine protease. β-Bergamotene (ΔG = −4.2044) indicated the greatest docking with trypsin proteinase. The ADMET evaluation of pure substance under examination demonstrated that β-bergamotene, α-eudesmol, β-farnesene, linalool, 1,8-cineole, eugenol, and α-guaiene affirmed ADMET descriptions of the highest quality, as measured by in-silico analyses [44].

Through the study of a molecule’s orientation or position on a possible target, molecular docking may be used to make predictions about the binding affinities and dynamics between the molecules. Putting the chemicals in a docking onto serine proteinases, metalloprotease, cysteine, and elastase protease exhibited several interactions, notably van der Waals interactions, H-bonds, and H-pi hydrophobic bonds. These bond interactions aided in comprehending the biological roles of numerous compounds in various fields, including pharmaceuticals and insecticides [44,45].

As per LC_50_ results, the *O. basilicum*’s volatile extract was active versus *R. ferrugineus*. The volatile extract exhibited clear insecticidal, antifeedant properties, and suppressed proteinases extracted from midgut preparation from the fourth instar. These findings shed light on substances that could be used to construct biochemical markers that reflect the insect resistance of specific plant varieties.

## 4. Materials and Methods

### 4.1. Chemicals, Reagents, and Media

The chemical solutions, acids, solvents, media, media chemicals, reagents, α-guaiene, and β-caryophyllene for biochemical studies were purchased from Sigma-Aldrich Co., St. Louis, MO, USA. Pure compounds of gallic acid, γ-terpinene, estragole, linalool, β-farnesene, 1,8-cineole, eugenol, β-terpineol, β-bergamotene, and nerol were supplied by Merck Co., St. Louis, MO, USA. Germacrene D was supplied by Aobious Inc., 9 Blackburn Drive, Gloucester, MA 01930, USA. α-Eudesmol was provided by BioCrick BioTech, 88 Keyuan Road, Hi-Tech Zone, Chengdu, Sichuan 610042, China.

### 4.2. Plant Materials

The *O. basilicum* seed was procured from a commercial nursery in the eastern region of Saudi Arabia. Seeds were decontaminated and seeded in an MS medium involving agar and sucrose at pH = 5.8, with 0.6 and 3% (*w*/*w*), respectively. The medium was cultured for 7–8 weeks, with seedling lengths of 19–20 cm in a climatic chamber at the optimum conditions of 26 ± 3 °C, 16 h light at King Faisal University, Saudi Arabia.

### 4.3. O. basilicum Callus and Cell Suspension Initiation with V. dahliae as a Biotic Elicitor

For this experiment, we sterilized, delinted, and cultivated *O. basilicum* seeds in Petri dishes with sterile blotted paper at a temperature of 28 ± 1 °C with a light intensity of 30 Einsteins/(m^2^.s) [26]. The *O. basilicum*’s explants (hypocotyls, cotyledonary, and epicotyls, length 4~6 mm) were seeded in an MS medium containing 0.1 mg/L 2,4-D, 0.5 mg/L kinetin, 1 mg/L IBA, and 0.5 mg/L NAA, as well as sucrose (3% *w*/*v*) as per Darrag et al., with changes [26]. Plant growth regulators were absent from the control treatment. Sub-cultures were started every three weeks while all cultures were maintained in a climate chamber (16 h, light, and 26 ± 3 °C) for seven weeks. Callus growth enhancement was evaluated using *V. dahliae* as an initiator. Callus was removed from individual cultures utilizing vacuum filtration 72 h after infection, and for 40 days, Callus was given a visual assessment every five days. Sub-cultivation of the *V. dahliae* occurred every 35 days in PDA (at 21 °C). For ten days, conidia were cultivated on potato dextrose (PD) medium at 22 degrees Celsius using a 240 rpm rotary shaker. After centrifugation, three washes were performed with a 0.1 M K_2_HPO_4_-KH_2_PO_4_ solution at a pH of 6.5 to remove any remaining debris from the conidia. Under a microscope, conidia concentration was measured with a hemocytometer. We injected either 25 µL of conidial suspension ((3–5) × 10^7^ conidia/mL) or control (sterile water) into 8 mL of freshly prepared MS solid medium.

### 4.4. Total Protein Assay in O. basilicum’s Callus and Cell Suspension

As per Darrag et al., with changes [26], using LS media, callus was initiated and detected for 56 weeks. The medium was passed via filters with various mesh sizes. A conidial suspension ((2–5) × 10^7^ conidia/mL) was added to 25 mL of LS culture medium (200 mL total volume), transmitted to 30 flasks (Erlenmeyer, 500 mL), and calibrated to a 250 mL final volume utilizing the liquid medium. After 72 h, the protein content of the cultures was evaluated. (250 mL) conical flasks with agar-free LS medium (100 mL) were used to produce suspension media, which was then maintained in a climate chamber for six weeks at 30 ± 2 °C, 16 h light circumstances, and shaking at 110 rpm, with subculture every fourteen days.

Protein content increases in cell suspension and callus were determined every five days until the callus was 40 days old. At intervals of 5–40 days of cell suspension, cultures were collected 72 h after inoculation to identify protein content. Protein estimation was carried out according to the method of Bradford [37].

In brief, aliquots of protein extract were placed in clean test tubes (protein concentration was evaluated at intervals ranging from 5 to 40 days after callus and cell suspension formation). Mix one hundred microliters of protein extract and 1 mL of working solution, add distilled water (3 mL), give the mixture a good shake, and let it sit for 2 min at 25 °C. The optical density was evaluated spectrophotometrically at 595 nm; the protein concentration was recorded using a standard curve of BSA. The standard curve was established by using different concentrations of BSA (5–100 µg), similar to that described above.

### 4.5. Characterization Using GC-MS Analysis

After 40 days, the cell suspension hydro distillate extract was diluted with n-hexane, employing an auto-sampler injector and administrated (1 μL) with an automatic injector (GC grade, 2 μL: 1 mL) (Model automatic sampler: Varian, Combi Pal). The GC-MS was used to detect that connection (GC, CP-3800, Walnut Creek, Varian, CA, USA, and MS, Varian, Saturn 2200). Its parameters were 0.25 μm thickness, 5% phenyl- dimethylpolysiloxane, 30 m length, and 0.25 mm inside diameter, with VF-5ms, a column of fused silica capillaries [9]. The ionization energy of the electron impact (EI) used in the ionization detector was 70 eV. The carrying gas was helium, with a constant flow rate of 1 mL/min maintained throughout the experiment. The transfer line and injector temperatures were 300 and 240 °C, respectively. For the first minute, the oven was maintained at 50 degrees Celsius, then for the next 50 min, it was heated to 230 degrees Celsius at a rate of 30 degrees Celsius per minute, then for the next 5 min, it was heated to 290 degrees Celsius at a rate of 10 degrees Celsius per minute, and finally, it was kept at isothermal conditions for 6 min. The total injection duration was 54.3 min, and the specimen injection split ratio was 1/500. The Wiley and NIST electronic libraries were used to develop a compound standard for n-alkanes (C6–C26) and determine the identities of the individual components.

### 4.6. Evaluation of the Extracted Secondary Metabolites’ Contact-Insecticide and Antifeedant Efficacy against R. ferrugineus

Final methanolic volatile extract concentrations were prepared by serial dilution (1, 10, 50, 100, 500, 1000, 5000, and 7000 g/mL). Moreover, compounds (estragole, (E)-β-ocimene, 1,8-cineole, β-terpineol, α-guaiene, eugenol, β-bergamotene, β-farnesene, germacrene D, α-eudesmol, nerol, linalool, and γ-terpinene) were dissolved in acetone and then added to 0.1% TritonX-100 [26]. Cells were suspended in the methanolic extract at serially increasing concentrations for 40 days. To dilute the compounds, TritonX-100 (0.1%) was added to acetone after the chemicals were dissolved. Adults or larvae of *R. ferrugineus* were purchased. They were then cultured using long lengths of sugar cane stem. The extracts’ anti-larvae action was assessed by applying topically to larvae and keeping them at 4–5 °C for 5 min. Utilizing a manually controlled micro-applicator, each larva had extract injected into its dorsum in three separate 10 µL doses using a 50-ll micro-syringe from Hertfordshire, UK’s Burkard Manufacturing Co., Ltd. (MS-N50; Shizuoka, Japan’s Ito Corp.). Five larvae in a box with three replications were fed on portions of long sugarcane stem measuring 10 cm. Larval mortality was assessed 24, 48, 72, and 96 h after topical medication to calculate the LD_50_. The sugarcane stem’s ten-centimeter-long portions cut into equal longitudinal halves were used to measure the antifeedant activity against adults. Long sections of sugarcane stem with a surface area of approximately 32.2 cm^2^ were dried in air at room temperature, assuming a dipping time of 10 s and an initial serial concentration of (10 mL). Each treated object remained in a plastic container. Each box included an additional pair (male and female). Each therapy was administered ten times. In total, 24-, 48-, 72-, and 96-h feeding observations were assessed.

### 4.7. Assessment of O. basilicum Cell Suspension Extract and Pure Components on R. ferrugineus Larvae’s Overall Proteolytic Enzyme Activity (In Vitro)

Protein concentrations were measured utilizing the Lowry approach [46]. The homogenate of *R. ferrugineus* 4th midgut instar larvae (lab strain) was assayed for total proteolytic enzyme activity using azocasein, as Darrag et al. [26], with modifications. Ten 4th midgut larval homogenates were carefully removed, incised, and then rinsed in a solution containing 0.9% NaCl before even being homogenized in a buffer (500 µL) containing 5 mM dithiothreitol (DTT), 0.1% (*v*/*v*) Triton X-100, and 50 mM N-2-hydroxyethyl piperazine-N′-2-ethane sulphonic acid (HEPS), and 8.0 pH. It was spun in a centrifuge (Sigma 3k30), and the remaining homogenates were from a preliminary phase for 30 min at 5000 rpm. Using the supernatants, the protein concentration and proteolytic enzyme activity were determined. A total, including assay buffer (60 µL), was used to incubate the supernatant (10 µL) at 37.5 °C for 20 min before azocasien (200 µL, 2% (*w*/*w*)) was added (pH = 8). Before each reaction experiment, 40-day-old methanolic extract of cell suspension, pure chemicals, and 10 μL of enzyme specimens were incubated for 10 min. The addition of the substrate initiated the reaction, which was allowed for 20 min in the case of leupeptin before being terminated 180 min later by adding 300 µL of cold tri-chloroacetic acid (TCA) (10%, *v*/*v*). Twenty minutes were spent centrifuging the mixture at 5000 rpm (Sigma 3k30). Overall, 10 µL of NaOH (10 N) was added to the supernatant, and the absorbance was measured using an ELISA plate reader at 450 nm to quantify the enzymes’ specific activity as OD450/mg protein/h.

### 4.8. Assessment of the Cell Suspension Extract and Components on Serine Proteinase (In Vitro) Specific Activity of R. ferrugineus Larvae

We used a serine protease assay buffer and a modified version of the fast microplate method to analyze the serine proteinase’s specific activity in mixtures (150 µL) [26]. After 30 min of centrifugation (8000 rpm), the test buffer (100 mM Tris-HCl) was removed from the midgut homogenate of fourth instar larvae (pH 8.1) (Rotors, Model No. 12158, Sigma 3K30) [26]. The sample (10 µL) was diluted using an assay buffer to 50 µL to determine the presence of trypsin proteinase, chymotrypsin proteinase, and elastase. Assay buffer was used to dilute stock substrates of BAρNA (100 mg/mL DMSO), both SAAPFρNA and SAAPLρNA (100 mg/mL DMF), to 1.0 mg/mL. To halt the reaction after 15 min of incubation at 37 °C, 50 µL of acetic acid (30%, *v*/*v*) was added. The intensity of the activity was measured using a 405 nm ELISA plate reader. A denaturation enzyme was used in place of an active enzyme in the experiment mixture. For three substrates (SAAPLρNA, BAρNA, and SAAPFρNA), activity levels of certain proteinases were assessed as OD mg^−1^ protein min^−1^.

### 4.9. Assessment of the Cell Suspension Extract and Pure Components on the Specific Activity of Metalloproteinase (In Vitro) of R. ferrugineus Larvae

The 4th midgut instar larval homogenate was used to test metalloproteinases activity using the substrate CEGR (DABCYL-Cys-Glu-Gly-Arg-Ser-Ala-EDANS-NH2) [26], with modifications. The ten midgut larvae from the fourth stage were neatly eliminated, dissected, and repeatedly washed with 0.9% NaCl solution. A 500 µL protease test solution, including 50 mM HEPS, 0.1% Triton X-100, and 5 mM DTT (pH 8.0), was utilized to homogenize the midgut instar. The homogenates were then centrifuged employing a Sigma 3k30 (cooling centrifuge) at 5000 rpm for 30 min to evaluate the supernatant’s enzyme activity and protein content. Before introducing CEGR (125 µL, 2% (*w*/*v*)), each experiment’s supernatant (10 µL) was incubated in an assay buffer (60 µL) at 37 °C for 20 min (pH 8). During a 10 min incubation period without a substrate, 10 µL enzyme samples were tested (20 min. for EDTA, EGTA, 1.10, Phenanthroline). After 180 min at 37 °C, 300 µL of cold TCA, 10% (*v*/*v*), was used to terminate the reaction. For the next 20 min, the mixture was spun (5000 rpm, Sigma 3K30). The absorbance was measured at 450 nm after mixing the supernatant with hydroxide solution (10 µL, 10 N) using an ELISA reader. An enzyme-free assay combination was utilized as a blank, and particular activity was evaluated utilizing the OD493 mg^−1^ protein min^−1^ and an enzyme-free blank specimen.

### 4.10. Assessment of Cell Suspension Extract and Components on Cysteine Proteinase Activity of R. ferrugineus Larvae (In Vitro)

Utilizing the substrate N-benzoyl-Phe-Val-Arg-*p*-nitroanilide hydrochloride and certain modifications, the cysteine proteinases of *R. ferrugineus* homogenate from the fourth midgut instar was tested for activity. Ten larvae of the fourth midgut were carefully taken out, dissected, and periodically washed with 0.9% (*w*/*v*) NaCl solution, assay buffer containing 50 mM HEPS, 5 mM DTT, and 0.1% Triton X-100 (*v*/*v*) (pH 8.0, 500 µL) was used to homogenize the samples. The homogenates were spun using a cooling Sigma 3k30 centrifuge for 30 min at 5000 rpm. The supernatant was tested and found to have a verifiable amount of protein and an active form of all the proteolytic enzymes. Before introducing 100 µL of the substrate, 10 µL of the supernatant was incubated in assay buffer (60 µL, pH = 8) at 37 °C for 30 min. Enzyme samples in 10 µL were incubated for 10 min with iodoacetic acid before the addition of the substrate. We added mersalyl (1.5 mL, 5 mM), garnet rapid reactive solution (0.02 mg/mL), and Tween 20 (2%, *v*/*v*) to terminate the reaction at 37.5 degrees Celsius after 60 min. The absorbance at 520 nm was measured after spinning the reaction for 6 min at 5000 rpm. The activity in the midgut was assayed at OD520.60 min^−1^ mg^−1^ of protein with an ELISA rapid reader.

As a starting point (blank), an assessment combination not containing enzymes was utilized. TLCK (trypsin proteinase inhibitor), PMSF (general serine proteinase inhibitor), EDTA (EDTA-metalloproteinase inhibitor), iodoacetic acid (an iodoacetic acid-cysteine proteinase inhibitor), TPCK (chymotrypsin-proteinase inhibitor), and Leupeptin (as general proteinase inhibitor) were added to the reactions. Homogenates of larval fourth-gut tissue were prepared using the same methodology described for the enzyme activity test. The inhibitory test was conducted via a microplate assay, as reported previously. A series of inhibitor levels were prepared to determine the greatest inhibition activity. Concentrations of 0.01, 0.05, 0.1, and 1.0 mM leupeptin; 0.005, 0.01, 0.05, 0.1, and 1 mM TLCK and TPCK; 0.1, 1.0, 10, 50, and 100 mM PMSF; and 0.01, 0.05, 0.1, 1, and 2 mM iodoacetic acid, 100 mM; 1,10 phenanthroline, EDTA, and EGTA concentrations of 0.1, 1, 10, 50, and 100 mM.

Various inhibitors were applied to understand the proteases found in the larval fourth of instars’ homogenate midgut preparations of *R. ferrugineus*. In all protease activity assay studies, 10 µL of enzyme samples specimens were treated with a pre-incubation period of extract or pure substances for 10 min (estragole, β-bergamotene, (E)-β-ocimene, β-terpineol, α-guaiene, 1,8-cineole, eugenol, germacrene D, β-farnesene, α-eudesmol, nerol, linalool, and γ-terpinene) or inhibitors at concentrations of 1, 10, 50, 100, 500, 1000, 5000, and 7000 mg/L. The impact of previous concentrations of each enzyme’s activity in the presence of an extract or substances in treated larvae was assessed in vivo after a 24-h treatment period. An experiment that was set up free of potential inhibitors served as a control. An ELISA plate fast reader measured the enzymes’ absorption at various wavelengths. For each inhibitor, the control enzyme’s percentage activity was assessed for each enzyme. Four instars midgut larvae were treated with previous concentrations of extracts or compounds, assessed as previously mentioned, to measure the protease activity in vivo. Larvae were subjected to extract- and compound-treated leaf discs for 24 h.

### 4.11. Docking of Experimented Compounds into Enzymes

Using the protein data bank (PDB), we obtained serine- (PDB:3F7O) [47], metallo- (PDB:1KAP) [48], cysteine- (PDB:3IOQ) [44], and trypsin proteinase (PDB:1FN8] [49], which was thereafter transferred to the Molecular Operating Environment (MOE). To compensate for the lack of hydrogen, heteroatoms and crystallographic molecules of water were eliminated from the protein’s chemistry [50]. The compounds (estragole, β-terpineol, (E)-β-ocimene, α-guaiene, β-bergamotene, germacrene D, eugenol, 1,8-cineole, β-farnesene, α-eudesmol, nerol, linalool, and γ-terpinene) were drawn using Chem Draw professional 15 builder module. After reducing the number of ligands, creating three-dimensional structures, removing duplications, and inserting bonds, the molecule was ready for docking. After inputting the default configuration and producing structures with the minimum energy, the ligands were made elastic and manually positioned within the catalytic site cavity presented in the enzyme model. The MOE 2015.10 (Molecular Operating Environment (MOE), Montreal, QC, Canada) was utilized for the protein–ligand docking, and a caused fit approach was used, which indicates that the receptor is stiff and the ligand is elastic [46]. The full-force field was used to determine the binding energy and scoring techniques that estimated free-binding interaction energies using terms from the molecular force field to calculate the affinity between the ligand and the protein. After obtaining the docking data, we recorded calculations and RMSD values and confirmed that the optimal ligand interaction had been achieved.

### 4.12. ADMET Screening

To evaluate the tested pure compounds’ toxicity risks (estragole, β-terpineol, (E)-β-ocimene, α-guaiene, β-bergamotene, germacrene D, eugenol, 1,8-cineole, β-farnesene, α-eudesmol, nerol, linalool, and γ-terpinene), they were subjected to using ADMETLAB 2.0 for in silico ADMET analysis. The ADMET collection consists of components and data on the tested substances’ toxicity. Intestinal absorption, aqueous solubility, hydrogen bond acceptor (HBA), distribution coefficient P (LogP), hydrogen bond donor (HBD), solubility (LogS), blood–brain barrier (BBB), human hepatotoxicity (H-HT), cytochrome P450 (CYP450), and plasma protein binding (PPB) were utilized to anticipate ADMET characteristics for all compounds. The models employed in this approach to anticipate ADMET attributes are gathered from numerous experimental data sources and product documentation.

### 4.13. Statistical Design

Statistics and probit analysis were performed using SPSS 25.0, as mentioned by Finney [51]. To sum up, all numerical predictions of toxicity variables and findings were presented as the mean ± SE. We regressed mortality and translated the resulting dosage to an LC_50_ value (µg/mL). Using the relative growth rate’s least-squares regression analysis (control %) with the extract level’s logarithm, the range of LC_50_ was determined with a confidence interval of 95 percent. Enzyme activity data were analyzed using an ANOVA. Means were separated using the SNK (Student–Newman–Keuls) test, and deviations at the *p* ˂ 0.05 level were considered noteworthy.

## 5. Conclusions

The findings suggested that *R. ferrugineus* could be controlled by using volatile secondary metabolites extracted from *O. basilicum* volatiles extracts as a bio-insecticide. The volatile secondary metabolites are from *O. basilicum*’s cell suspension extract. β-Bergamotene, α-eudesmol, β-farnesene, linalool, 1,8-cineole, eugenol, α-guaiene, and β-caryophyllene had a significant insecticidal action versus *R. ferrugineus*’ adults. α-Eudesmol, linalool, 1,8-cineole, eugenol, and nerol revealed the greatest objective application: activities versus larvae. The research discovered a correlation between the production of volatile secondary metabolites and their application, which may be related to the terpenoids and polyphenolic compounds in basil. Determining the effectiveness and impacts of such secondary metabolites requires extensive research. Analysis of molecular docking and in silico ADMET were performed, as well as the potential for developing environmentally friendly bio-insecticides. Additionally, cell suspension can be used to make these secondary metabolites in large quantities. This straightforward, clean technique allows for the investigation of the created compounds and their final formulation for use.

## Figures and Tables

**Table 1:** *O. basilicum*’s cell suspension extract’s chemical components classes and the overall identified extract, including oxygenated sesquiterpenes (OS), oxygenated monoterpenes (OM), sesquiterpene hydrocarbons (SH), monoterpene hydrocarbons (MH), overall phenylpropanoids (PP).

No.	Compounds	RI (exp)	RI (lit)	Relative Abundance %
	Monoterpene Hydrocarbons (MH)			
**1**	α-thujene	929	924	0.1± 0.10
**2**	α-pinene	937	932	0.1 ± 0.02
**3**	camphene	952	946	0.1± 0.02
**4**	sabinene	973	973	0.4± 0.06
**5**	β-pinene	977	977	1 ± 0.02
**6**	β-myrcene	991	988	0.1 ± 0.04
**7**	α-phellandrene	1005	1002	0.1 ± 0.04
**8**	car-4-ene	1009	1004	1.2 ± 0.1
**9**	α-terpinene	1017	1014	0.1 ± 0.1
**10**	limonene	1030	1224	0.4 ± 0.1
**11**	(Z)-β-ocimene	1038	1032	0.2 ± 0.02
**12**	(*E*)-β-ocimene	1049	1044	11.96 ± 0.2
**13**	γ-terpinene	1060	1067	1.0 ± 0.2
**14**	terpinolene	1088	1086	0.7 ± 0.1
**Total Monoterpene Hydrocarbons (MH) Identified %**				**17.46 ± 1.12**
	**Oxygenated Monoterpenes (OM)**			
**1**	1,8-cineole (eucalyptol)	1031	1031	7.24 ± 0.4
**2**	linalool (β-linalool)	1099	1095	1.2 ± 0.2
**3**	β-terpineol	1130	1130	12.37 ± 0.87
**4**	camphor	1145	1141	1.4 ± 0.2
**5**	borneol (isoborneol)	1167	1165	0.2 ± 0.05
**6**	terpinen-4-ol	1177	1174	2 ± 0.3
**7**	α-terpineol	1189	1186	0.1 ± 0.04
**8**	estragole	1199	1199	22.38 ± 0.7
**9**	fenchyl acetate	1214	1214	0.1 ± 0.02
**10**	nerol	1228	1227	1.6 ± 0.2
**11**	neral	1244	1244	0.3 ± 0.1
**12**	*p*-mentha-1,8-dien-7-ol	1261	1261	0.1 ± 0.04
**13**	bornyl acetate	1285	1284	0.5 ± 0.02
**14**	methyl geranate	1321	1319	0.3 ± 0.03
**15**	neryl acetate	1364	1359	0.1 ± 0.04
**Total Oxygenated Monoterpenes (OM) Identified as %**				**49.89 ± 2.88**
	**Sesquiterpene Hydrocarbons (SH)**			
**1**	α-copaene	1376	1374	0.4 ± 0.03
**2**	(*E*)-β-bourbonene	1384	1387	0.1 ± 0.02
**3**	α-cubebene	1385	1387	0.5 ± 0.02
**4**	β-cubebene	1389	1387	0.7 ± 0.05
**5**	β-elemene	1391	1389	0.1 ± 0.03
**6**	7-epi-sesquithujene	1402	1405	0.3 ± 0.04
**7**	α-gurjunene	1409	1409	0.1 ± 0.02
**8**	β-caryophyllene	1424	1424	1.1 ± 0.1
**9**	β-copaene	1432	1430	0.2 ± 0.05
**10**	β-gurjunene (calarene)	1434	1431	0.2± 0.04
**11**	*trans*-α-bergamotene	1435	1432	0.2 ± 0.03
**12**	α-guaiene	1439	1437	4.8 ± 0.2
**13**	β-bergamotene	1441	1438	2.3 ± 0.2
**14**	α-humulene	1455	1452	1.52 ± 0.04
**15**	(*E*)-β-farnesene	1457	1454	2.3 ± 0.08
**16**	*cis*-muurola-4(14),5-diene	1463	1465	0.2 ± 0.1
**17**	γ-muurolene	1477	1478	0.1 ± 0.03
**18**	germacrene D	1481	1484	4.2 ± 0.2
**19**	β-selinene	1486	1489	0.2 ± 0.03
**20**	β-bulnesene	1505	1508	0.1 ± 0.02
**21**	β-bisabolene	1509	1512	0.3 ± 0.06
**22**	γ-cadinene	1513	1513	0.7 ± 0.02
**23**	β-sesquiphellandrene	1524	1521	0.8 ± 0.03
**24**	δ-cadinene	1525	1522	0.4 ± 0.04
**25**	α-cadinene (α-amorphene)	1538	1537	0.1 ± 0.02
**Total Sesquiterpene Hydrocarbons (SH) Identified as %**				**21.92** ± 1.50
	**Oxygenated Sesquiterpenes (OS)**			
**1**	τ-cadinol	1640	1638	0.1 ± 0.02
**2**	α-eudesmol	1653	1652	1.8 ± 0.1
**3**	α-Bisabolene oxide	1680	1682	0.1 ± 0.02
**Total Oxygenated Sesquiterpenes (OS) Identified (%)**				**2.0 ± 0.14**
	**Phenylpropanoids (PP)**			
**1**	chavicol	1256	1247	0.1 ± 0.03
**2**	eugenol	1357	1356	3.7 ± 0.3
**3**	methyl eugenol	1406	1402	0.4 ± 0.02
**Total Phenylpropanoids (PP) Identified (%)**				**4.2 ± 0.35**
	**Non-Terpene Derivatives**			
**1**	ethyl isovalerate	853	856	0.3 ± 0.02
**2**	6-methyl-5-hepten-2-one	985	988	0.4 ± 0.02
**Total Non-Terpene Derivatives (NT) Identified (%)**				0.7 ± 0.04
Total Identified (%)				96.17

Values in Table 1 were gathered from triplicates (n = 3; mean ± standard deviation (SD)); RI (lit) relative retention index from MS libraries (Wiley); RI (exp): relative retention index established on capillary column (VF-5ms fused silica); National Institute of Standards and Technology (NIST).

**Table 2:** Probit and mortality analysis of *R. ferrugineus* adults and fourth larvae treated with pure compounds and *O. basilicum* volatile extract.

Extract	Adult				4th Larvae			
	LC_50_ (µg/mL) 95% CF	Slope	Chi-Square	*p*	LD_50_ (µg/Larvae) 95% CF	Slope	Chi-Square	*p*
***O. basilicum***	1229 (1041–1392)	2.54 ± 0.20	42.4	<0.01	13.8 (12.01–15.58)	1.29 ± 0.23	42.41	<0.01
**estragole**	3587 (2269–2847)	2.04 ± 0.22	49.1	0.009	34.8 (31.7–35.6)	1.08 ± 0.12	49.2	0.008
**β-terpineol**	3924 (2694–4259)	1.94 ± 0.23	49.7	0.009	42.6 (39.7–44.3)	1.04 ± 0.11	49.3	0.009
**(E)-β-ocimeme**	3014 (1845–2356)	2.12 ± 0.23	48.2	0.009	31.2 (27.4–32.8)	1.12 ± 0.13	48.2	0.008
**1,8-cineole**	1426 (1197–1594)	2.69 ± 0.19	43.9	0.006	13.9 (12.8–14.7)	1.36 ± 0.18	41.8	0.001
**α-guaiene**	1491 (1265–1682)	2.62 ± 0.21	44.8	0.007	19.2 (17.8–20.7)	1.24 ± 0.17	44.2	0.004
**germacrene D**	2045 (1825–2314)	2.48 ± 0.22	47.2	0.009	21.3 (19.7–22.5)	1.20 ± 0.17	44.6	0.006
**eugenol**	1459 (1226–1647)	2.66 ± 0.20	44.4	0.006	14.2 (13.1–15.4)	1.34 ± 0.19	42.2	0.002
**β-bergamotene**	1294 (1086–1418)	2.76 ± 0.21	43.2	0.003	17.2 (16.1–18.4)	1.28 ± 0.16	43.4	0.003
**β-farnesene**	1356 (1178–1572)	2.73 ± 0.20	43.7	0.004	18.4 (17.1–19.7)	1.26 ± 0.17	43.8	0.004
**α-eudesmol**	1312 (1167–1548)	2.75 ± 0.19	43.6	0.004	12.4 (11.3–13.1)	1.39 ± 0.17	41.3	0.001
**nerol**	1947 (1569–2119)	2.51 ± 0.22	46.2	0.009	15.6 (14.3–16.2)	1.31 ± 0.18	42.3	0.002
**linalool**	1398 (1176–1583)	2.71 ± 0.20	43.8	0.005	13.7 (12.6–14.8)	1.37 ± 0.18	41.6	0.001
**β-caryophyllene**	1523 (1312–1736)	2.60 ± 0.21	45.7	0.008	20.1 (18.7–21.4)	1.22 ± 0.17	44.3	0.005
**γ-terpinene**	3841 (2589–4027)	1.97 ± 0.22	49.4	0.009	38.3 (35.1–40.7)	1.07 ± 0.14	49.8	0.008

LD_50_: lethal dose; LC_50_: lethal concentration; CF: confidence limits; LC_50_ and LD_50_ with 95% CF. The data are given as the value ± standard error (SD) of five replicates for each concentration tested: *n* = 10.

**Table 3:** Compounds’ docking scores within the active sites of serine proteinase (PDB:3F7O), metalloproteinase (PDB:1KAP), trypsin proteinase (1FN8), and cysteine proteinase (PDB:3IOQ).

Compounds	Docking Score ΔG (kcal/mol)			
	Serine Proteinase	Cysteine Proteinase	Metallo Proteinase	Trypsin Proteinase
Estragole	−4.3709 (10)	−4.5151 (9)	−4.6842 (12)	−4.3164 (13)
β-terpineol	−4.1040 (12)	−4.6980 (7)	−4.6411 (13)	−4.4555 (10)
(E)-β-ocimeme	−4.5195 (6)	−4.4243 (13)	−4.9996 (9)	−4.5755 (9)
1,8-cineole	−4.0485 (14)	−4.4759(10)	−4.2706 (14)	−4.2897 (14)
α-guaiene	−4.4206 (7)	−4.8005 (6)	−5.3298 (4)	−4.8352 (5)
germacrene D	−4.4132 (9)	−4.4563 (11)	−5.4350 (3)	−4.5790 (8)
Eugenol	−4.9100 (3)	−4.6507 (8)	−4.8218 (10)	−4.3781 (12)
β-bergamotene	−4.9929 (2)	−5.0760 (2)	−5.3167 (5)	−5.2044 (1)
β-farnesene	−5.0728 (1)	−5.0141 (3)	−5.6725 (1)	−5.1448 (3)
α-eudesmol	−4.6450 (5)	−5.2551 (1)	−5.2549 (6)	−5.1964 (2)
nerol	−4.7396 (4)	−4.4430 (12)	−5.5082 (2)	−4.7201 (7)
linalool	−4.0872 (13)	−4.9738 (4)	−5.0587 (7)	−4.9127(4)
β-caryophyllene	−4.4190 (8)	−4.8315 (5)	−5.0288 (8)	−4.7619 (6)
γ-terpinene	−4.1591 (11)	−4.1835 (14)	−4.7142 (11)	−4.4256 (11)

## Data Availability

Data is contained within the article.

## Supplementary Materials

The following supporting information can be downloaded at: https://www.mdpi.com/article/10.3390/plants11212949/s1, Figure S1: GC-MS Chromatogram of *O. basilicum* cell suspension extract; Figure S2: In vitro specific inhibitors against protease enzymes in *R. ferrugineus* larval instars; Figure S3: Docking and ligand interactions of α-guaiene (A1, A2), β-caryophyllene (B1, B2), germacrene D (C1, C2), estragole (D1, D2), γ-terpinene (E1, E2), β-terpineol (F1, F2), linalool (G1, G2), and 1,8-cineole (H1, H2) within the active sites of serine proteinase (PDB:3F7O); Figure S4: Docking and ligand interactions of β-terpineol (A1, A2), eugenol (B1, B2), estragole (C1, C2), 1,8-cineole (D1, D2), germacrene D (E1, E2 nerol (F1, F2), (G1, G2) (E)-β-ocimene, and (H1, H2) γ-terpinene within the active sites of cysteine proteinase (PDB:3IOQ); Figure S5: Docking and ligand interactions of linalool (A1, A2), β-caryophyllene (B1, B2), (E)-β-ocimene (C1, C2), eugenol (D1, D2), γ-terpinene (E1, E2), estragole (F1, F2) β-terpineol, (G1, G2), and (H1, H2) 1,8-cineole within the active sites of metalloproteinase (PDB:1KAP); Figure S6: Docking and ligand interactions of nerol (A1, A2), germacrene D (B1, B2), (E)-β-ocimene (C1, C2), β-terpineol (D1, D2), γ-terpinene (E1, E2), eugenol (F1, F2), estragole (G1, G2), and 1,8-cineole (H1, H2) within the active sites of trypsin proteinase (PDB:1FN8); Table S1: Mean total protein concentration (µg/mL) using Bradford assay in *O. basilicum* from the callus of both infected and noninfected *V. dahliae* strain; Table S2: ADMET analysis of estragole (22.38%), β-terpineol (12.37%), (E)-β-ocimeme (11.96%), 1,8-cineole (7.24%), α-guaiene (4.8%), germacrene D (4.2%), Eugenol (3.7%), β-bergamotene (2.3%), β-farnesene (2.3%), α-eudesmol (1.8%), nerol (1.6%), α-humulene (1.52%), linalool (1.2%), and γ-terpinene (1%).
