# Supplementary material for: Analysis of Volatile Secondary Metabolites in Ocimum basilicum Cell Suspensions: Inhibition, In Silico Molecular Docking, and an ADMET Analysis against Proteolytic Enzymes of Rhynchophorus ferrugineus
Source: Plants (Basel). 2022 Nov 1;11(21):2949. doi: 10.3390/plants11212949 (PMC9655874; doi:10.3390/plants11212949)
Supplement: Supplementary file 1 [file plants-11-02949-s001.zip › plants-1988661-supplementary.pdf]

Supplementary materials

Table S1. Mean total protein concentration (µg/mL) using Bradford assay in *O. basilicum* from the callus of both infected and noninfected *V. dahliae* strain.

**Age of Callus or cell suspension Sample (days)**	***O. basilicum***			
	**µg protein/mL extract (1 g callus)**		**µg protein/mL extract (1 g cell suspension)**	
	Without Infection	With Infection	Without Infection	With Infection
**5**	68.41^{p}±0.38	203.88^{m}±0.62	12.73^{p}±0.42	20.70^{o}±0.43
**10**	118.95^{o}±2.02	271.54^{j}±0.50	31.49^{n}±0.68	79.21^{l}±0.38
**15**	179.50^{n}±0.52	339.95^{f}±0.50	72.01^{m}±0.70	145.20^{i}±0.38
**20**	227.60^{l}±1.38	398.88^{e}±0.40	110.69^{k}±0.51	195.16^{e}±0.49
**25**	267.59^{k}±0.54	448.96^{d}±0.43	127.71^{j}±0.60	224.93^{d}±0.43
**30**	283.88^{i}±0.40	479.01^{c}±0.50	146.66^{h}±0.68	245.87$_{c}$±0.40
**35**	300.10^{h}±0.65	498.58^{b}±1.49	156.87^{g}±0.30	263.97^{b}±0.57
**40**	305.24^{g}±0.48	514.25^{a}±0.45	165.42^{f}±0.30	275.12^{a}±0.43
F	75255		87019	
**Df**	15, 32		15, 32	

Data are mean ± SD of triplicates. In the same column, values labeled with the same letter are not significantly different from one another (P ≤ 0.0001 ≡ V.H.Sig. for all values) as per the SNK test (Student–Newman–Keuls). Df: freedom degree, F: F ratio, and P: p-value (the F ratio significance o).

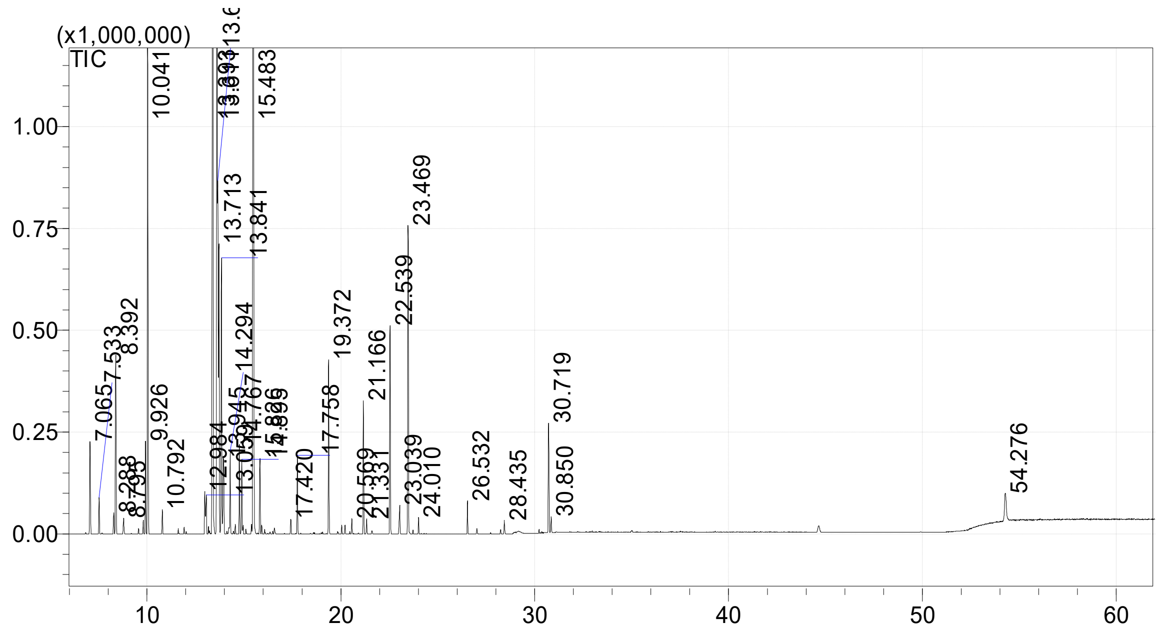

Figure S1. GC-MS Chromatogram of *O. basilicum* cell suspension extract.

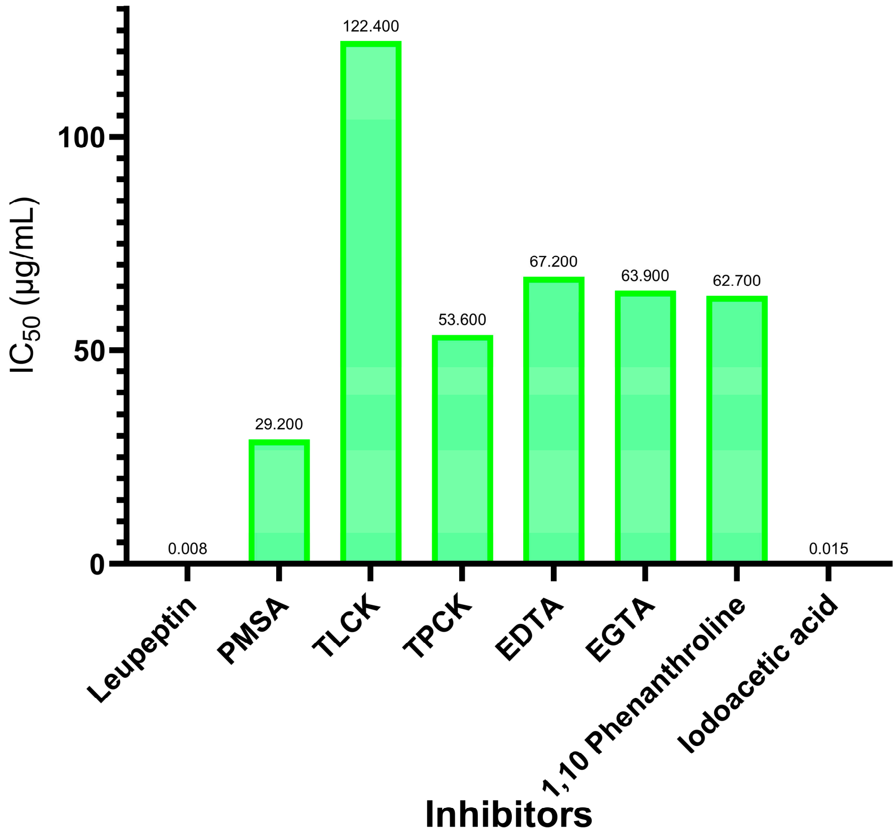

Figure S2. In vitro specific inhibitors against protease enzymes in *R. ferrugineus* larval instars; Leupeptin: general proteinase Inhibitor; PMSF: a general inhibitor of serine proteinase (elastase); TLCK: trypsin inhibitor; TPCK: chymotrypsin inhibitor; 1,10 phenanthroline, EGTA, and EDTA: metalloprotease inhibitor; iodoacetic acid: cysteine protease inhibitor.

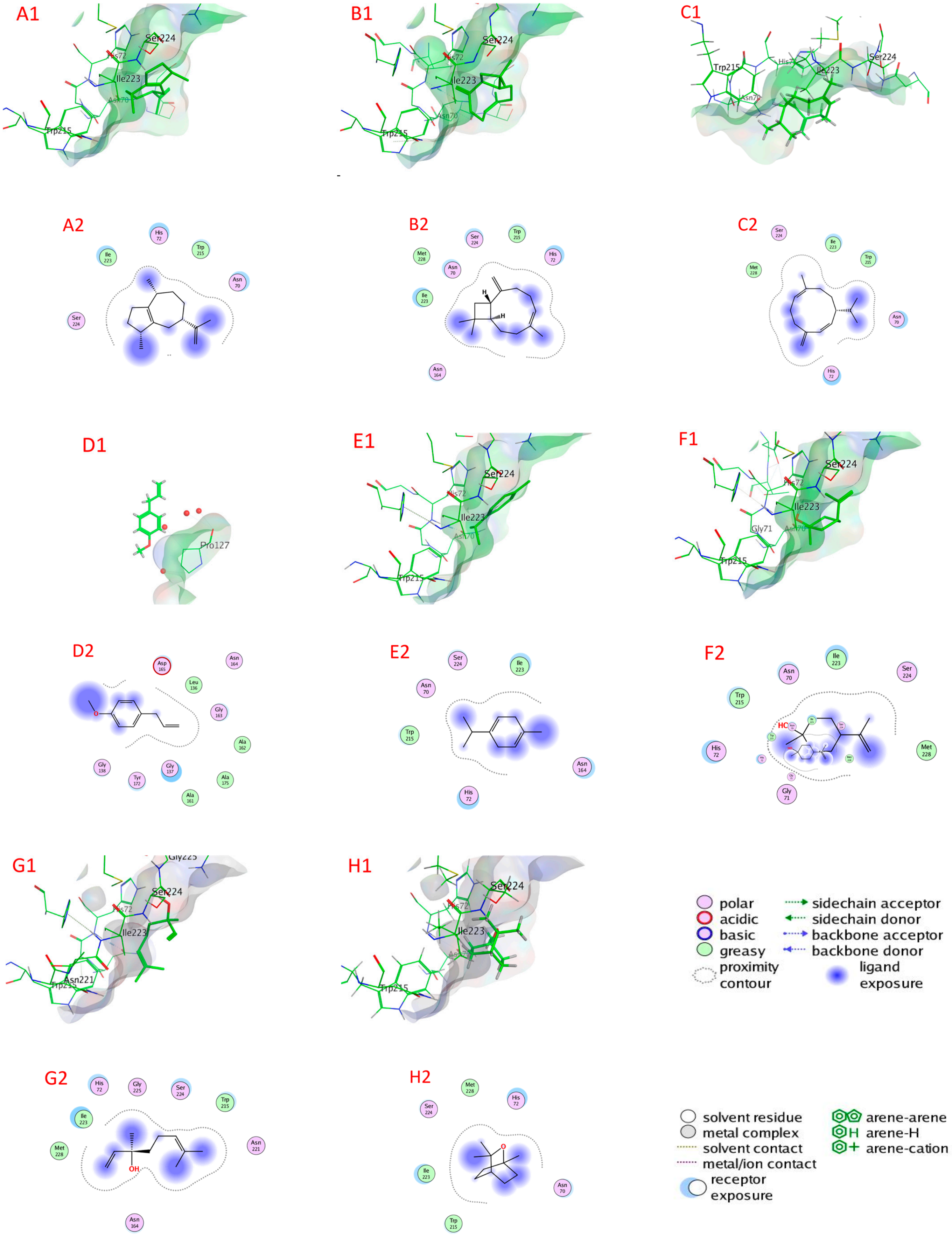
A1
Ser224
His72
Ile223
Asn70
Trp215
B1
Ser224
His72
Ile223
Asn70
Trp215
C1
Trp215
His72
Ile223
Ser224
Asn70
A2
B2
C2
D1
Pro127
E1
Ser224
His72
Ile223
Asn70
Trp215
F1
Ser224
His72
Ile223
Gly71
Asn70
Trp215
D2
E2
F2
G1
Gly225
Ser224
His72
Ile223
Asn221
Trp215
H1
Ser224
His72
Ile223
Trp215
G2
OH
H2
polar
acidic
basic
greasy
proximity contour
sidechain acceptor
sidechain donor
backbone acceptor
backbone donor
ligand exposure
solvent residue
metal complex
solvent contact
metal/ion contact
receptor exposure
arene-arene
arene-H
arene-cation

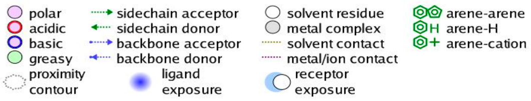

Figure S3. Docking and ligand interactions of α-guaiene (A1, A2), β-caryophyllene (B1, B2), germacrene D (C1, C2), estragole (D1, D2), γ-terpinene (E1, E2), β-terpineol (F1, F2), linalool (G1, G2), and 1,8-cineole (H1, H2) within the active sites of serine proteinase (PDB:3F7O).

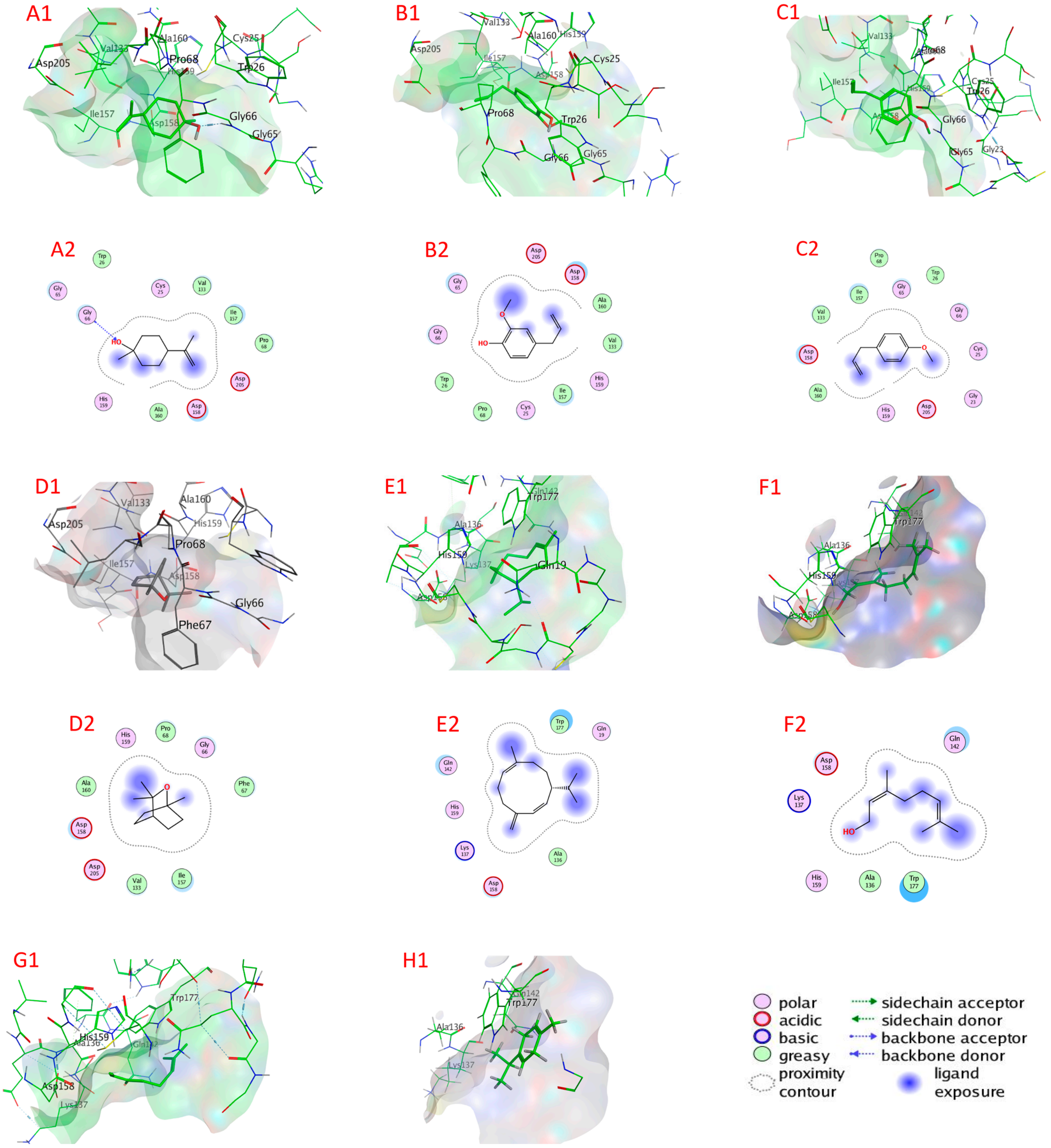

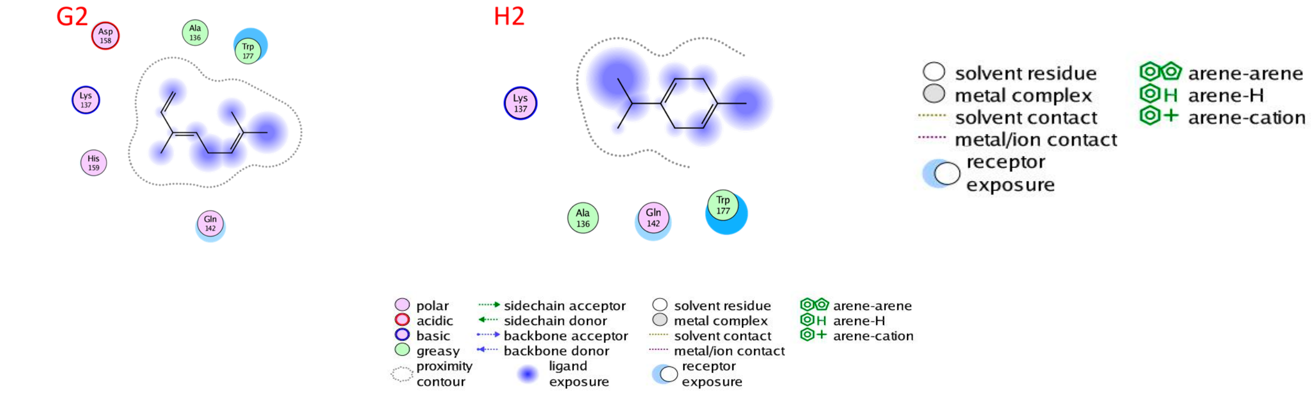

Figure S4. Docking and ligand interactions of β-terpineol (A1, A2), eugenol (B1, B2), estragole (C1, C2), 1,8-cineole (D1, D2), germacrene D (E1, E2 nerol (F1, F2), (G1, G2) (E)-β-ocimene, and (H1, H2) γ-terpinene within the active sites of cysteine proteinase (PDB:3IOQ).

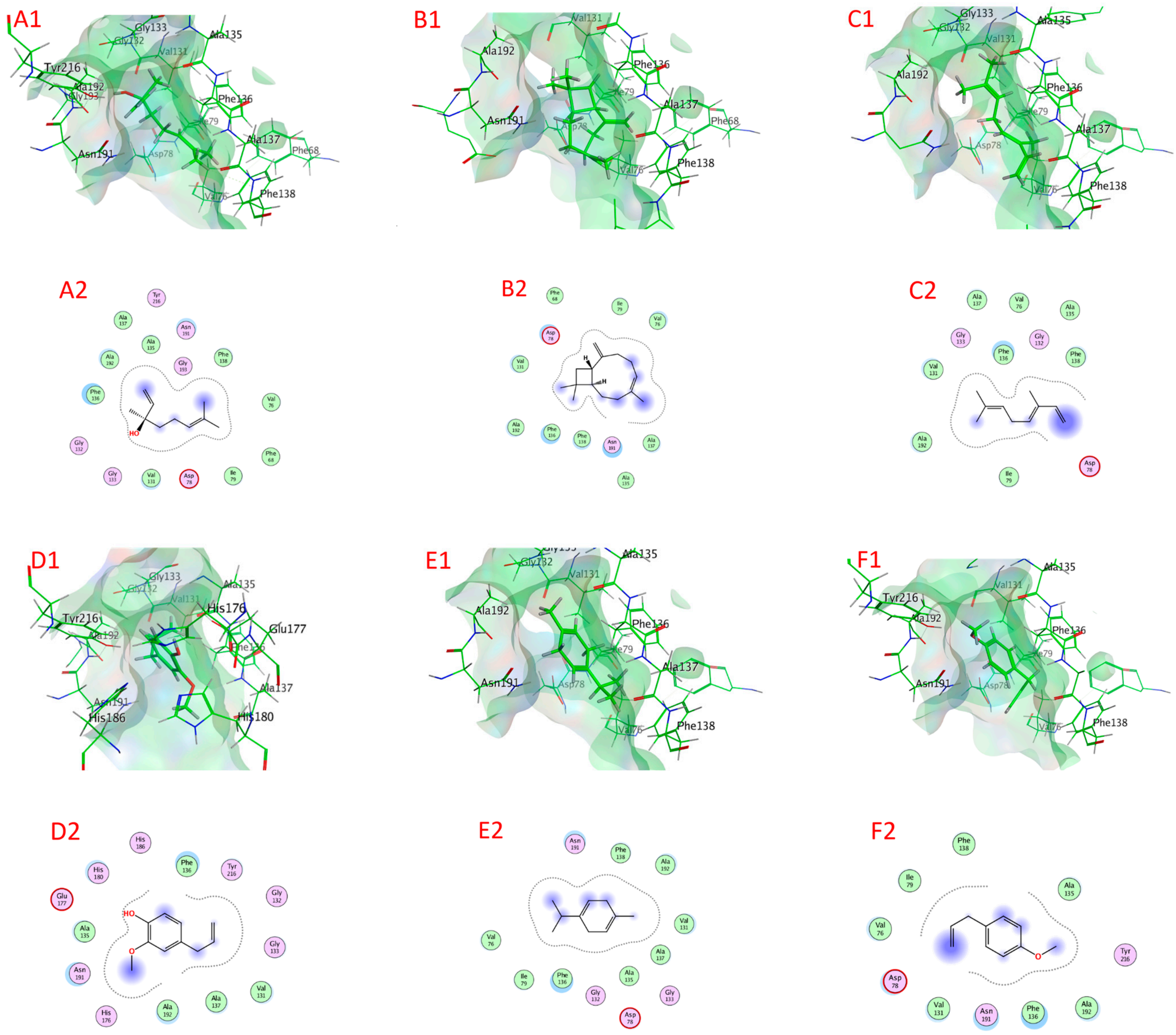

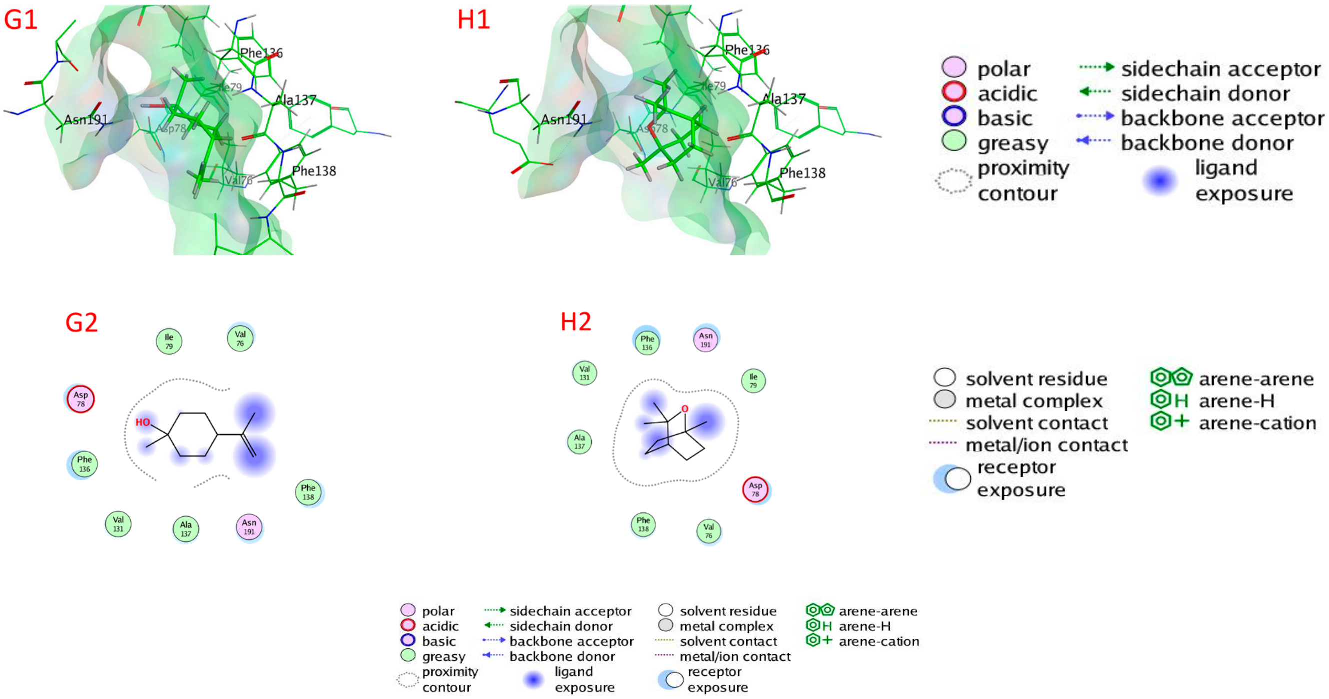

Figure S5. Docking and ligand interactions of linalool (A1, A2), β-caryophyllene (B1, B2), (E)-β-ocimene (C1, C2), eugenol (D1, D2), γ-terpinene (E1, E2), estragole (F1, F2) β-terpineol, (G1, G2), and (H1, H2) 1,8-cineole within the active sites of metalloproteinase (PDB:1KAP).

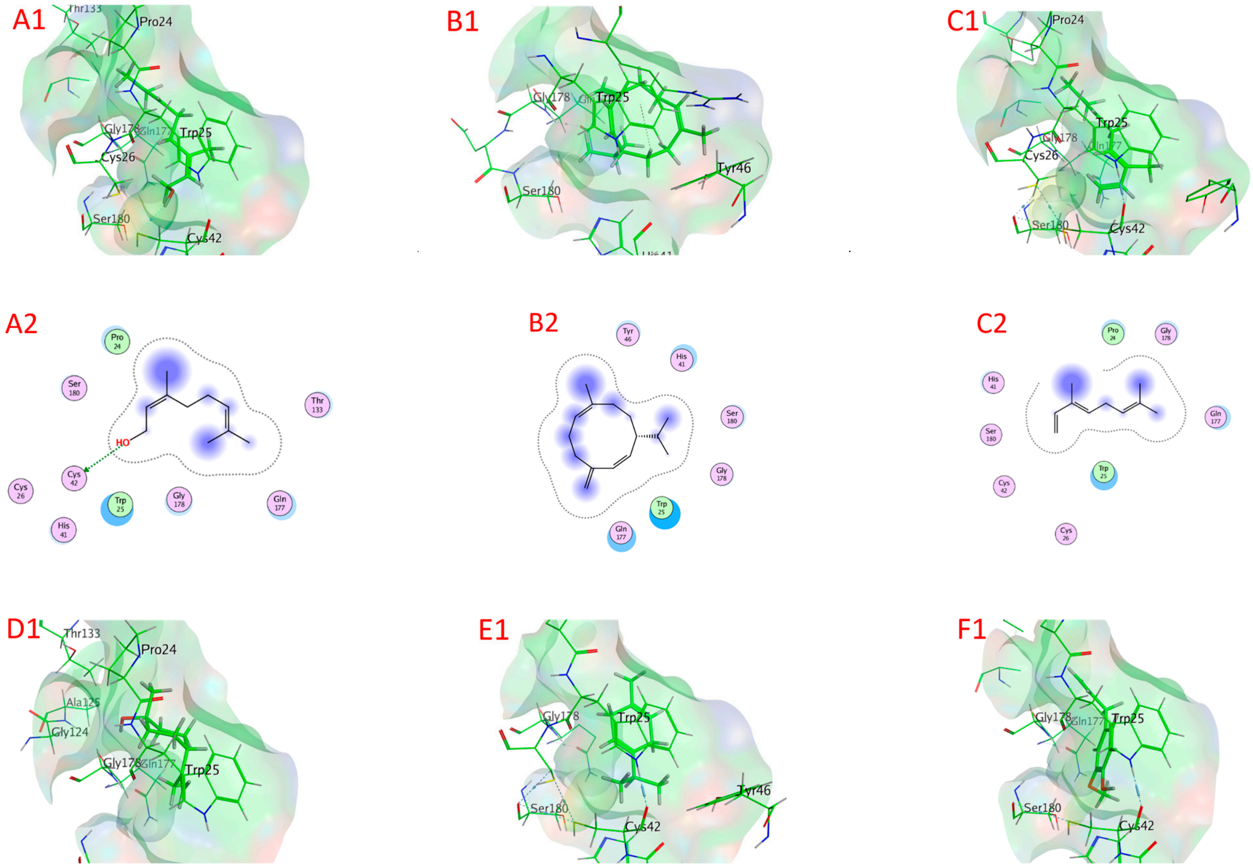

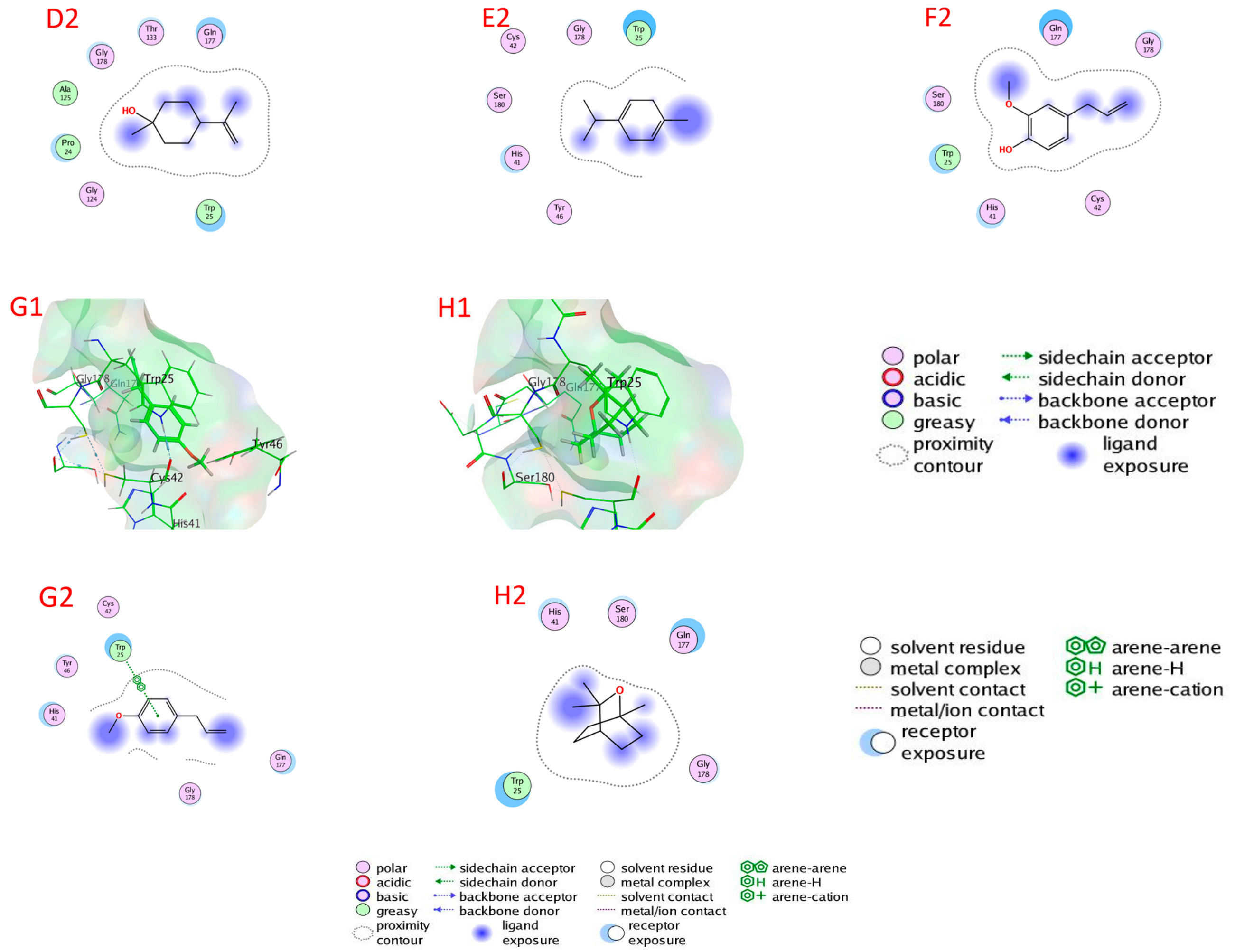

Figure S6. Docking and ligand interactions of nerol (A1, A2), germacrene D (B1, B2), (E)-β-ocimene (C1, C2), β-terpineol (D1, D2), γ-terpinene (E1, E2), eugenol (F1, F2), estragole (G1, G2), and 1,8-cineole (H1, H2) within the active sites of trypsin proteinase (PDB:1FN8).

Table S2. ADMET analysis of estragole (22.38%), β-terpineol (12.37%), (E)-β-ocimeme (11.96%), 1,8-cineole (7.24%), α-guaiene (4.8%), germacrene D (4.2%), Eugenol (3.7%), β-bergamotene (2.3%), β-farnesene (2.3%), α-eudesmol (1.8%), nerol (1.6%), α-humulene (1.52%), linalool (1.2%), and γ-terpinene (1%).

**Compounds**	**HBD**	**HBA**	**LogP**	**LogS (μg/mL)**	**BBB**	PPB	**CYP450 2D6 substrate**	H-HT	TPSA
Estragole	0	1	2.424	264.157 μg/mL	+++	+	+++	-	9.23
β-terpineol	1	1	2.504	1783.338 μg/mL	+++	+	-	-	20.23
(E)-β-ocimeme	0	0	3.475	6.114 μg/mL	+++	+	+	-	0
1,8-cineole	0	1	2.744	1703.074 μg/mL	+++	+	-	---	9.23
α-guaiene	0	0	4.725	0.269 μg/mL	+++	+	-	---	0
germacrene D	0	0	4.891	0.11 μg/mL	+++	+	-	---	0
Eugenol	1	2	2.129	2225.275 μg/mL	+++	+	+++	---	29.46
β-bergamotene	0	0	4.725	0.259 μg/mL	+++	+	-	---	0
β-farnesene	0	0	5.202	0.175 μg/mL	+++	+	+	---	0

α-eudesmol	1	1	3.92	14.794 μg/mL	+++	+	-	-	20.23
nerol	1	1	2.671	266.216 μg/mL	++	+	-	-	20.23
linalool	1	1	2.67	1099.597 μg/mL	+++	+	-	-	20.23
β-caryophyllene	0	0	4.725	0.275 μg/mL	+++	+	-	---	0
γ-terpinene	0	0	3.309	7.84 μg/mL	+++	+	+	-	0

HBA: hydrogen bond acceptor; HBD: hydrogen bond donor; LogS: Solubility; LogP: distribution coefficient P; PPB: plasma protein binding; BBB: blood-brain barrier; H-HT: human hepatotoxicity. LogS: optimal: higher than -4 log mol/L, LogS <10 μg/mL: low solubility; LogS 10–60 μg/mL: moderate solubility; LogS >60 μg/mL: high solubility; LogP: optimal: 0< LogP <3, LogP <0: poor lipid bilayer permeability, LogP >3: poor aqueous solubility; BBB: BB ratio >=0.1: BBB+; BB ratio <0.1: BBB-; classification of models & probability 0~0.1 (---), 0.1~0.3 (--), 0.3~0.5 (-), 0.5~0.7 (+), 0.7~0.9 (++), and 0.9~1 (+++).
